# iPS Cell-Based Model for *MAPT* Haplotype as a Risk Factor for Human Tauopathies Identifies No Major Differences in TAU Expression

**DOI:** 10.3389/fcell.2021.726866

**Published:** 2021-08-31

**Authors:** Tabea Strauß, Amir Marvian-Tayaranian, Eldem Sadikoglou, Ashutosh Dhingra, Florian Wegner, Dietrich Trümbach, Wolfgang Wurst, Peter Heutink, Sigrid C. Schwarz, Günter U. Höglinger

**Affiliations:** ^1^German Center for Neurodegenerative Diseases (DZNE), Munich, Germany; ^2^Department of Neurology, Technical University Munich, Munich, Germany; ^3^German Center for Neurodegenerative Diseases (DZNE), Tübingen, Germany; ^4^Department of Neurology, Hanover Medical School, Hanover, Germany; ^5^Center for Systems Neuroscience, Hanover, Germany; ^6^Institute of Developmental Genetics, Helmholtz Zentrum München, Oberschleißheim, Germany; ^7^TUM School of Life Sciences, Technical University of Munich, Freising, Germany; ^8^Department for Neurodegenerative Diseases, Hertie Institute for Clinical Brain Research, University of Tübingen, Tübingen, Germany; ^9^Geriatric Clinic Haag, Haag in Oberbayern, Germany

**Keywords:** TAU, α-SYNUCLEIN, iPSC, disease modeling, NGN2 neurons, *MAPT* haplotype

## Abstract

The H1 haplotype of the microtubule-associated protein tau (*MAPT*) gene is a common genetic risk factor for some neurodegenerative diseases such as progressive supranuclear palsy, corticobasal degeneration, and Parkinson’s disease. The molecular mechanism causing the increased risk for the named diseases, however, remains unclear. In this paper, we present a valuable tool of eight small molecule neural precursor cell lines (smNPC) homozygous for the *MAPT* haplotypes (four H1/H1 and four H2/H2 cell lines), which can be used to identify *MAPT*-dependent phenotypes. The employed differentiation protocol is fast due to overexpression of *NEUROGENIN-2* and therefore suitable for high-throughput approaches. A basic characterization of all human cell lines was performed, and their TAU and α-SYNUCLEIN profiles were compared during a differentiation time of 30 days. We could identify higher levels of conformationally altered TAU in cell lines carrying the H2 haplotype. Additionally, we found increased expression levels of α-SYNUCLEIN in H1/H1 cells. With this resource, we aim to fill a gap in neurodegenerative disease modeling with induced pluripotent stem cells (iPSC) for sporadic tauopathies.

## Introduction

A major class of neurodegenerative diseases are tauopathies which are characterized by intra-cellular inclusions of the microtubule-associated protein tau (*MAPT*) in neurons. TAU is encoded by the *MAPT* gene on chromosome 17q21 and can be spliced into six isoforms by inclusion or exclusion of exons 2, 3, and 10 (Goedert and Jakes, 1990). Alternative splicing of exon 10 results in isoforms with either three or four microtubule-binding repeats (3R or 4R-TAU; Goedert et al., 1989). According to the dominant isoform detected in the brain inclusions, tauopathies can be classified as 3R, 4R, and 3R/4R tauopathies (Sergeant et al., 2005). A classical 3R tauopathy is Pick’s disease (PiD), while progressive supranuclear palsy (PSP) and corticobasal degeneration (CBD) are typical 4R tauopathies (Dickson, 1998; Dickson et al., 2002, 2010). Primary age-related tauopathies (PART) and Alzheimer’s disease (AD) display deposits with both isoforms (Goedert et al., 2006; Crary et al., 2014). Over 50 known mutations in the *MAPT* gene causing familial forms of frontotemporal lobar degeneration with TAU inclusions (FTLD-TAU; Ghetti et al., 2015) include missense mutations that alter the amino acid sequences of TAU and splice mutations which influence the alternative splicing of exon 10 (Goedert, 2005). Apart from these autosomal dominant mutations, there are several genetic risk factors that increase the risk for neurodegenerative diseases like tauopathies.

After a first association of a polymorphic dinucleotide marker between exon 9 and 10 in the *MAPT* gene with PSP (Conrad et al., 1997), thorough analysis revealed a complete linkage disequilibrium of multiple polymorphisms in this gene, defining two extended haplotypes H1 and H2. Spanning not only the whole TAU gene (Baker et al., 1999) but a region of approximately 1.8 Mb on chromosome 17q21, the *MAPT* haplotype is one of the largest haplotypes in the human genome (Ezquerra et al., 1999; Pastor et al., 2002; Pittman et al., 2004; Caffrey and Wade-Martins, 2007). Characteristic for the H2 haplotype is a nearly 1 Mb long inversion, which includes the *MAPT* gene next to other genes (Stefansson et al., 2005). The H1 haplotype was repeatedly connected to a higher risk for developing primary tauopathies such as PSP and CBD and the secondary tauopathy AD (Myers et al., 2005; Höglinger et al., 2011; Kouri et al., 2015). Surprisingly, genome-wide association studies identified the *MAPT* haplotype also as major risk factor for Parkinson’s disease (PD), which is not considered a tauopathy but a synucleinopathy (Pascale et al., 2016). Additionally, Vuono et al. (2015) reported a connection between the rate of cognitive decline and the *MAPT* haplotype in Huntington’s disease.

The molecular mechanisms of the H1 haplotype causing an increased risk for developing a sporadic neurodegenerative disease is still under investigation. Haplotype defining single nucleotide polymorphisms (SNPs) are mainly found in intronic regions, not altering the amino acid sequence of TAU (Kwok et al., 2004). Whether the *MAPT* haplotype influences the total TAU expression or only the expression of one specific TAU isoform is still subject to debate (Kwok et al., 2004; Caffrey et al., 2006; Myers et al., 2007; de Jong et al., 2012; Trabzuni et al., 2012; Majounie et al., 2013; Valenca et al., 2016).

So far, most studies have only investigated the haplotype-dependent mRNA expression, but not its translation into TAU protein. Additionally, there is the need for a human cell model to investigate the mechanisms behind the *MAPT* haplotype. Induced pluripotent stem cell (iPSC)-derived neurons have become a valuable tool for investigating tauopathies. The ability to convert somatic cells from individuals with specific genetic variants into human neurons has proven an appealing instrument. Missense and intronic mutations in the *MAPT* gene that are connected to FTLD-TAU were shown to cause differences in mRNA and protein expression of TAU in iPSC-derived neurons (Imamura et al., 2016; Garcia-Leon et al., 2018; Verheyen et al., 2018). Familial forms of tauopathies are only representing a small percentage of cases, therefore justifying an urgent need to model genetic risk factors for sporadic tauopathies like the *MAPT* haplotype. While a recent iPSC model used H1/H2 heterozygous cell lines to investigate allele-specific *MAPT* mRNA expression (Beevers et al., 2017), a systematic comparison of homozygous H1 and H2 cell lines is missing. With the presented iPSC resource, we attempt to close that gap.

## Materials and Methods

### Identification and Selection of iPSC Lines

Commercially available cell lines from healthy donors from the ECACC cell bank, specifically from the HipSci collection (Human Induced Pluripotent Stem Cell Initiative),^1^ were selected regarding their *MAPT* haplotype, passage number and pluripotency score, as well as their donors’ age and sex (see Table 1). The openly accessible genome sequencing data of healthy control individuals was searched and filtered for three haplotype defining SNPs: rs1800547, rs8070723, and rs1052553. 4 H1/H1 and 4 H2/H2 cell lines were purchased (for genotyping of further SNPs and microsatellite Rep1 see Tables 2–6). For simplicity, all cell lines are referred to only by their four-letter code in the end of the name (e.g., yemz for HPSI1013i-yemz_1). For all cell lines an MTA was signed from our lab, as well as all collaborating partners on this project.

**TABLE 1:** General information of used human iPS cell lines.

Cell line name^a^	*MAPT* haplotype^b^	Donor age^c^	Donor sex	PPS^d^	nPPS^e^	Passage number iPS cells	ECACC^f^ catalog number
HPSI1013i-yemz_1	H1/H1	70–74	Male	29.15	1.41	21	77650060
HPSI0913i-diku_1	H1/H1	60–64	Female	28.09	1.26	28	77650088
HPSI0614i-lepk_1	H1/H1	60–64	Female	29.90	1.30	13	77650367
HPSI0115i-melw_2	H1/H1	60–64	Male	22.83	1.30	20	77650564
HPSI0115i-zihe_1	H2/H2	75–79	Female	24.11	1.23	19	77650561
HPSI0614i-uilk_2	H2/H2	55–59	Male	8.5	1.50	14	77650606
HPSI0114i-zapk_3	H2/H2	60–64	Male	34.18	1.30	22	77650156
HPSI1113i-qolg_1	H2/H2	35–39	Male	54.55	0.86	21	77650145

*^*a*^In future reference, cell lines are referred to only by their four-letter code in the end of the name (e.g., yemz for HPSI1013i-yemz_1).*

*^*b*^Used SNP for genotyping: rs1800547, rs8070723, and rs1052553.*

*^*c*^Donor age is only given as a range by European Collection of Authenticated Cell Cultures.*

*^*d*^Pluripotency score.*

*^*e*^Novel pluripotency score.*

*^*f*^European Collection of Authenticated Cell Cultures.*

**TABLE 2:** Single nucleotide polymorphisms (SNPs) defining the H1 sub-haplotype.

Sub-haplotypes	rs1467967	rs242557	rs3785883	rs2471738	rs8070723	rs7521
H1b	G/G	G/G	G/G	C/C	A/A	A/A
H1c	A/A	A/A	G/G	T/T	A/A	G/G
H1d	A/A	A/A	G/G	C/C	A/A	A/A
H1e	A/A	G/G	G/G	C/C	A/A	A/A

### Genotyping

The selected cell lines were further analyzed regarding their genotype for SNPs in the *MAPT* gene and in the *SNCA* gene coding for α-SYNUCLEIN. The genotyping array results for imputed and phased genotypes from each cell line, available at the HipSci website,^2^ were analyzed with the freely available software GenomeBrowse from Golden Helix (Bozeman, MT, United States) to identify SNP genotypes. Furthermore, for investigation of a microsatellite near the *SNCA* gene, first the GATK haplotype calls from the whole genome sequencing (also available on the HipSci website) were first viewed with the GenomeBrowse for identification of the genotype in the region of interest. Secondly, the raw sequencing reads of the whole genome sequencing data were viewed with the UCSC Browser^3^ to identify the whole sequence of the microsatellite.

### Induced Pluripotent Stem Cells Culture and smNPC Generation

Purchased iPS cells were thawed and expanded on Matrigel (Corning, NY, United States) in “Essential 8 Flex” medium (Thermo Fisher, Waltham, MA, United States). iPSCs were converted according to a modified protocol into small molecule neural precursor cells (smNPC) via embryoid body formation (Reinhardt et al., 2013; Dhingra et al., 2020). iPSC were detached and transferred into a low binding 6 well suspension plate with KSR medium containing 78% DMEM/F12 Medium, 20% Knockout serum replacement, 1% Non-essential amino acids, and 1% GlutaMax (Life Technologies, Carlsbad, CA, United States) and 0,1% β-Mercaptoethanol (Sigma-Aldrich, St. Louis, MO, United States) supplemented with 10 μM Dorsomorphine (Abcam, Cambridge, United Kingdom), 10 μM SB431542 (Selleckchem, Houston, TX, United States), 0.5 μM Purmorphamine (Biomol, Hamburg, Germany), 3 μM CHIR99021 (Tocris, Bristol, United Kingdom) and 2 μM Thiazovivin (Millipore, Billerica, MA, United States). At day 2, the medium was changed to N2B27 medium consisting of 48.425% DMEM/F12 Medium, 48% Neurobasal Medium, 0.5% N2-supplement, 1% B27 supplement without Vitamin A (Life Technologies, Carlsbad, CA, United States), 0.025% Insulin (Sigma-Aldrich, St. Louis, MO, United States), 0.5% Non-essential amino acids, 1% GlutaMax, 1% Penicillin/Streptavidin (Life Technologies, Carlsbad, CA, United States) and 0.05% β-Mercaptoethanol supplemented with 10 μM Dorsomorphine, 10 μM SB431542, 0.5 μM Purmorphamine and 3 μM CHIR99021. At day 4, the medium was changed to smNPC expansion medium composed of N2B27 medium with 0.5 μM Purmorphamine, 3 μM CHIR99021 and 64 μg/ml ascorbic acid (Th. Geyer, Renningen, Germany). 6-day old embryoid bodies were mechanically disrupted by pipetting with a 1 ml filter tip and plated in a 6 well plate coated with Geltrex (Life Technologies, Carlsbad, CA, United States). The cells were passaged with Accutase (Sigma-Aldrich, St. Louis, MO, United States) when reaching a confluency of 80%. Thereafter, cells were passaged several times every 5–7 days until the culture contained compact smNPC colonies (seven and eight passages). At this point, cells were frozen for a cell stock of pure smNPCs.

### Differentiation Into Neurons

For rapid differentiation of smNPCs into neurons, the inducible lentivirus expression construct for the human transcription factor *NEUROGENIN-2* (pLV_TRET_hNgn2_UBC_Blast_T2A_rtTA3) was introduced into the smNPCs (Menden et al., 2021). The vector contains *NGN2* under control of the tetracycline operator and a blasticidin resistance. SmNPCs were plated at a cell density of 40,000/cm^2^ in a 12 well plate. The next day, the medium was refreshed containing 1 μM Thiazovivin and an hour later the virus was added. After 24 h, the cells were washed, and the medium was replaced with fresh expansion medium. 2–4 days later, cells were passaged and treated with 15 μg/ml Blasticidin (Invivogen, San Diego, CA, United States) for 15 days to select for the stable integration of the *NGN2* vector. After the selection step, cells were frozen to create a cell stock from which cells were thawed when fresh *NGN2*_smNPCs were needed.

**FIGURE 1:**
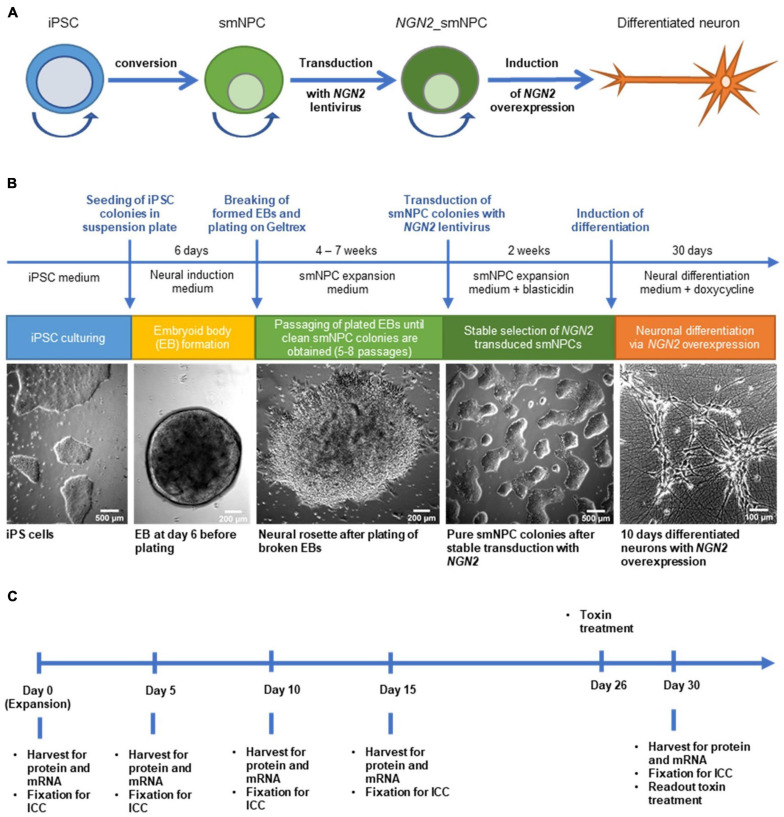
Generation of *NGN2* inducible small molecule neural precursor cell lines (smNPC) from induced pluripotent stem cells (iPSC) and experimental timeline. **(A)** Schematic protocol overview. iPSC were converted into smNPC via embryoid body formation. Obtained smNPC colonies were transduced with *NGN2*-lentivirus to generate *NGN2*_smNPC which can be differentiated into neurons upon *NGN2* overexpression. Round arrows indicate freezable and expandable cell populations. **(B)** Flow diagram of protocol procedure to generate differentiated neurons from iPSC. **(C)** Experimental timeline for differentiating neurons. Cells were harvested or fixed at five different time points for analysis.

To induce neuronal differentiation for experiments, *NGN2*_smNPCs were seeded on multi-well-plates sequentially coated with 100 μg/ml Poly-L-ornithine (Sigma-Aldrich, St. Louis, MO, United States) and 10 μg/ml Laminin (Sigma-Aldrich, St. Louis, MO, United States). Cells were directly seeded in induction medium consisting of N2B27 medium supplemented with 2.5 μg/ml doxycycline (Sigma-Aldrich, St. Louis, MO, United States) and 2 μM DAPT (Cayman, Ellsworth, United States). At day 3 of differentiation, the medium was completely changed to differentiation medium containing N2B27 medium with 2.5 μg/ml doxycycline, 10 μM DAPT, 10 ng/ml BDNF, 10 ng/ml GDNF, 10 ng/ml NT 3 (Peprotech, Princeton, NJ, United States) and 0.5 μg/ml laminin. From day 6 on, half of the medium was refreshed every 3 days by differentiation medium without doxycycline and DAPT. Cells were differentiated for several time periods and then harvested or fixed or the according readout was performed (see Figure 1C).

### Immunocytochemistry

Cells were seeded at a cell density of 30,000/cm^2^ in a 48 well plate on cover slips. The plates were fixed at different time points with 4% paraformaldehyde (Roth, Karlsruhe, Germany) for 10 min at room temperature and then washed twice with PBS. For immunofluorescent stainings, the cells were permeabilized with 0.2% triton-X in PBS. After washing twice with PBS, the cells were blocked in 1% BSA, 2% horse and goat serum, and 0.05% Tween in PBS for 1 h. Primary antibodies were diluted in blocking solution and incubated over night at 4°C (see Supplementary Table 1 for list of antibodies). The next day, the cells were washed twice with PBS-T and treated with secondary antibodies also diluted in blocking solution. After 2 h, cells were washed twice with PBS-T, then incubated for 10 min with DAPI (Thermo Fisher, Waltham, MA, United States; 1:1,000 in PBS) and washed with PBS. The cover slips were fixed on object slides with fluorescent mounting medium (Agilent Technologies, Santa Clara, CA, United States). The staining was analyzed using the Leica DMI6000B microscope (Leica Microsystems, Wetzlar, Germany) with a 40x air objective. Images were processed with the ImageJ software (Schindelin et al., 2012, 2015) and the brightness and contrast was set to the same values in all pictures from one staining. Cells were counted using the Cell Counter in the ImageJ software.

### Quantitative Real-Time PCR

Cells were seeded at a cell density of 50,000/cm^2^ in a 6 well plate and harvested at different time points with RLT lysis buffer from the RNeasy plus mini kit (Qiagen, Hilden, Germany). mRNA was isolated according to the manufacturer’s instructions. For gene expression analysis by semi-quantitative real-time PCR (qPCR), cDNA was prepared from 500 ng mRNA per reaction with the iScript kit (Bio-Rad Laboratories, Hercules, CA, United States). The reverse transcription was performed according to the manufacture’s protocol. 5 ng of the complementary cDNA and 0.2 μM forward and reverse primers together with the SYBR green select qPCR supermix (Thermo Fisher, Waltham, MA, United States) were used for the qPCR reaction which was then run using the StepOne Plus system (Applied Biosystems, Waltham, MA, United States). Sequences of the utilized primers are listed in Supplementary Table 2. The following reaction protocol was applied: 2 min at 50°C, 2 min at 95°C, and 40 cycles of 15 s at 95°C and 60 s at 60°C. Afterward, the melting curve was recorded. The cycle threshold (Cτ) values represent cycle numbers at which reaction reaches exponential phase. A cDNA standard serial dilution was prepared from a pool of sample cDNA and was run for each primer on every plate. Relative mRNA expression levels from samples were calculated with this relative standard curve to ensure comparability of samples between plates. As internal control, three housekeeping genes were measured (UBQLN1, PSMC1, and GPBP1).

### Western Blot

Cells were seeded in 6 well plates at a density of 50,000/cm^2^. At different time points, cells were harvested with M-PER lysis buffer (Thermo Fisher, Waltham, MA, United States) supplemented with protease inhibitors (cOmplete, Roche Basel, Switzerland) and phosphatase inhibitors (phoSTOP, Roche). The samples were incubated on ice for 15 min and then frozen at −80°C for at least 1 h to facilitate lysis of cells. After thawing, samples were incubated on ice for 15 min and then centrifuged at 2000 × *g* for 30 min. The supernatant containing the M-PER soluble protein fraction was transferred into a fresh tube and the protein concentration was measured with the BCA protein assay kit (Thermo Fisher, Waltham, MA, United States) with a BSA standard curve.

For insoluble protein extraction with sarkosyl, the remaining pellet was washed once with M-PER buffer and centrifuged at 45,000 rpm for 1 h. The supernatant was discarded, and the sarkosyl-buffer was added (1% sarkosyl, 10 mM Tris–HCl, 150 mM NaCl, and 5 mM EDTA). The pellet was disrupted by pipetting up and down, vortexing and was incubated at room temperature with shaking at 700 rpm. The samples were again ultracentrifuged at 45,000 rpm for 1 h and the supernatant (sarkosyl-soluble fraction) was collected as insoluble protein fraction. The protein concentration was measured as described before.

20 μg of protein were denatured with XT-buffer (95°C for 5 min) and loaded on 10% Bis-Tris Criterion polyacrylamide gels (Bio-Rad Laboratories, Hercules, CA, United States) and electrophoresis was run with MES running buffer at 150 V for 70 min (for detecting 4R TAU, 60 μg of protein was loaded). The protein transfer was carried out with a semidry blotting system (Trans-Blot Turbo Transfer System, Bio-Rad Laboratories, Hercules, CA, United States). Proteins were blotted on a 0.2 μm PVDF membrane for 40 min at 1 A and 25 V with transfer buffer containing 20% Methanol. After the transfer, the membrane was fixed in 0.4% paraformaldehyde for 20 min. After washing 3× with PBS, the membrane was blocked with 5% skimmed milk (Sigma-Aldrich, St. Louis, MO, United States) in TBS supplemented with 0.05% Tween-20 (Sigma-Aldrich, St. Louis, MO, United States; TBS-T) for at least 1 h and then incubated overnight at 4°C with the primary antibodies diluted in 1% BSA in TBS-T. The membrane was washed and incubated with secondary HRP-conjugated antibodies (diluted in 5% skimmed milk in TBS-T) for 1–2 h at room temperature. The blots were developed with Clarity Western ECL Substrate (Bio-Rad Laboratories, Hercules, CA, United States) or femto ECL (Thermo Fisher, Waltham, MA, United States) and the signal was detected with Odyssey Fc imaging system (LI-COR Biotechnology, Lincols, Netherlands). After imaging, the blots were analyzed with the Image Studio^TM^ Software from LI-COR. The used primary antibodies are listed in Supplementary Table 3. The following secondary antibodies were used: HRP-coupled anti-mouse IgG (1:2,500, Vector Laboratories, Burlingname, CA, United States) and anti-rabbit IgG (1:5,000, Vector Laboratories, Burlingname, CA, United States).

For stripping, the membrane was rinsed after imaging with TBS-T and then incubated in stripping buffer for 20–30 min at 50°C. Thereafter, the membrane was washed thoroughly first with deionized water and then with TBS-T before blocking again with milk. The incubation with primary and secondary antibodies as well as the imaging was performed as described above.

Two protein standards were generated by pooling some microliter of samples from day 5 until day 30 from the MPER-soluble samples and the sarkosyl-soluble samples, respectively. The protein concentration was determined, and 20 μg protein was loaded on every SDS gel together with the samples of interest for the soluble and the insoluble fraction, respectively. Signals from every western blot were normalized to the signal from the protein standard. For 4R TAU western blots a separate protein standard was created only including samples from late differentiation time points to ensure a detectable signal. For every time point at least three independent replicates for each cell line were generated and analyzed via Western blot.

### Toxin Treatment

Cells were seeded at a density of 20,000/well in a clear 96 well plate. At day 26 of differentiation, cells were treated with the mitochondrial inhibitor Annonacin. For the treatment, the toxin was diluted to a final concentration of 800 nM in differentiation medium. The medium in the plate was completely removed and replaced with medium containing the toxin. Cells were cultivated for four more days without media change. On day 30 of differentiation, the cell viability was assessed using the fluorescent dye Calcein-red-orange (Life Technologies, Carlsbad, CA, United States) and the MTT assay. First, the Calcein staining was carried out. An end concentration of 250 nM of the Calcein dye was added to the medium and the cells were incubated at 37°C for 30 min. Thereafter, the medium was completely removed and replaced by PBS. The fluorescence intensity was measured with the fluorescence microplate reader CLARIOstar from BMG Labtech (Ortenberg, Germany) at an excitation wavelength of 577 nm and emission wavelength of 620 nm. After the measurement, the cells were treated with the MTT solution to a final concentration of 500 ng/ml and incubated for an hour at 37°C. The medium was removed, and the plate was frozen at −80°C for at least 1 h and then thawed again to ensure cell lysis. The formed salt was dissolved in DMSO and the adsorption was measured at 570 nm. Each cell line was normalized to their own untreated control to account for possible differences in cell density.

### Production of TAU Protein and Aggregates and TAU Uptake Experiment

Recombinant 2N4R TAU protein was produced as described before (Goedert and Jakes, 1990). In brief, TAU protein was expressed in *E. coli* BL21 under IPTG induction and extracted from bacterial cells by freeze-thawing cycle and sonication. The extract was purified by a boiling step followed by centrifugation and the supernatant was further purified by ion-exchange chromatography.

The aggregates were generated via incubation of TAU protein at 3 mg/ml (65 μM) in PBS pH 7.4 and 1 mM dithiothreitol in the presence of 130 μM Heparin (∼3,000 kDa, MP Biomedicals) and 1,500 rpm agitation for 36 h. To obtain pure fibrils, the aggregates were fractionated by using centrifugation at 100,000 × *g* for 30 min and subsequently to achieve oligomers, the supernatant was filtrated through a 100 kDa ultrafilter (Millipore). In order to fluorescently label TAU, monomers or aggregates were incubated for 1 h with 200 μM of ATTO488-NH-ester (ATTO-TEC, Siegen, Germany) at room temperature. The unbound probes were removed for monomers by using Bio-Spin 6 size exclusion spin columns (Bio-Rad Laboratories, Hercules, CA, United States) and for aggregates by three times washing with PBS after centrifugation and filtration. The concentration of the monomers and aggregates was measured with a BCA assay kit. The proteins were preserved in −80 after flash-freezing in liquid nitrogen for further experiments.

For the uptake assay, *NGN2*_smNPCs were cultured in clear bottom black 96 well tissue culture plates (PerkinElmer). Between day 8 and 10 of differentiation, cells were incubated with 500 nM labeled TAU monomers, oligomers or fibrils for 3 h. Then, the medium was changed and fluorobrite DMEM (Gibco) containing 0.05% Trypan Blue was added to quench the extracellular fluorescence signal. The fluorescence was measured by a 20 × 20 matrix using CLARIOStar microplate reader. Since the cell density of each line was slightly different, to compare the TAU uptake among various lines, we used the viable cell staining Calcein-green in different wells of the same line in parallel to the uptake assay. The overall fluorescence from TAU uptake was normalized to the Calcein fluorescence intensity.

### Statistical Analysis

All statistical data analyses were performed with GraphPad Prism 9.1.0 for Windows (GraphPad Software, San Diego, CA, United States).^4^ Data displaying *MAPT* haplotype groups are represented as mean ± SEM of *n* = 4 cell lines for each haplotype. When individual cell lines are displayed, data are represented as mean ± SEM of independent experiments. Data with two factors (e.g., time and genotype) were analyzed with two-way ANOVA followed by a *post hoc* Tukey’s test or Sidak’s test. Otherwise, an unpaired *t*-test was used. Specifications are given in each figure legend.

## Results

### Differentiation and Characterization of All Human iPS Cell Lines

We selected 8 iPS cell lines from healthy donors from the European Collection of Authenticated Cell Cultures (ECACC; Table 1) according to their *MAPT* haplotype (four H1/H1 and four H2/H2 lines), their passage number and pluripotency scores, as well as their donors’ age and sex. All iPS cell lines were converted into smNPC via embryoid body formation and dual SMAD inhibition (Figures 1A,B). Thereafter, we treated the smNPCs with *NEUROGENIN-2* (*NGN2*)-lentivirus and selected via antibiotic resistance for a stable integration (Dhingra et al., 2020). The obtained *NGN2*_smNPCs could be cryopreserved (master and working cell bank) or kept in expansion for several weeks (up to twelve passages). Differentiation was achieved by inducing the *NGN2* overexpression via doxycycline addition. For the experimental procedures, we differentiated cells for 5, 10, 15, and 30 days and expanding cells were used as day 0 reference (Figure 1C). The cells were characterized with different markers via immunocytochemistry.

**FIGURE 2:**
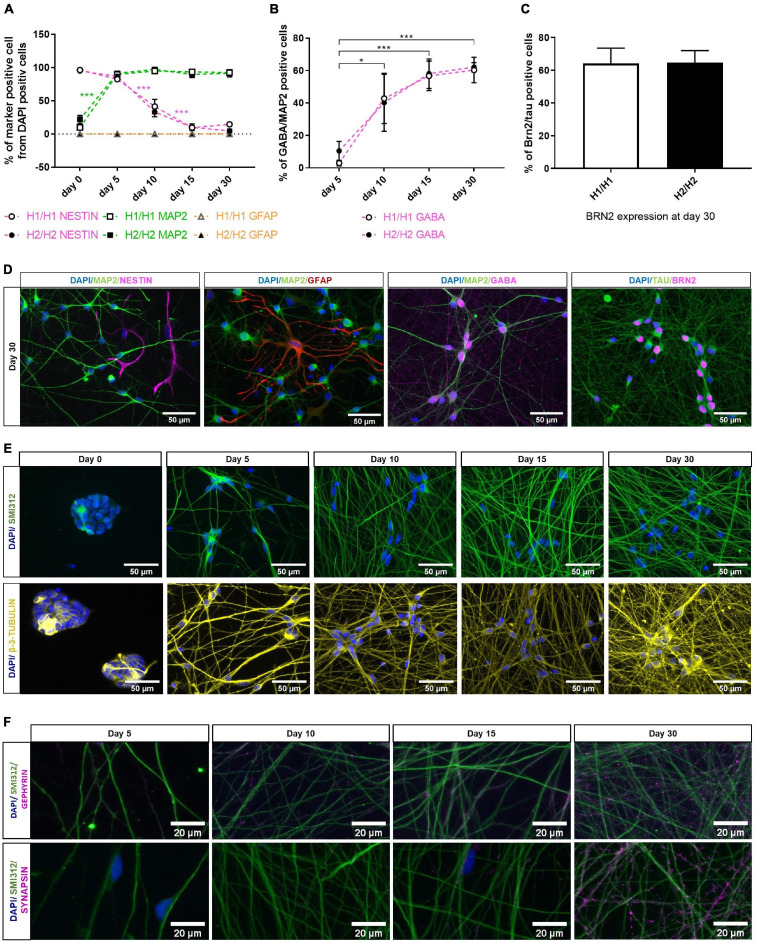
Characterization of differentiated neurons overtime with immunocytochemistry. **(A)** Quantification of different cell types with dendritic marker MAP2, neural progenitor marker NESTIN and glia marker GFAP over differentiation time of 30 days. MAP2 expression increases significantly upon differentiation, while percentage of NESTIN positive cells decreases. Glia cells are nearly absent from cell culture (under 1% at day 30). Data are represented as mean ± SEM of *n* = 4 cell lines for each haplotype group. Two-way ANOVA followed by Tukey’s test was performed. ***p < 0.001. **(B)** GABA expression is significantly upregulated during differentiation period. At day 30 of differentiation, 61% of neuronal cells (MAP2 positive cells) are GABAergic (GABA positive cells). Data are represented as mean ± SEM of *n* = 4 cell lines for each haplotype group. Two-way ANOVA followed by Tukey’s test was performed. *p < 0.05, ***p < 0.001. **(C)** 65% of TAU-positive neurons express marker for cortical layer 2/3 neurons (BRN2) at day 30 of differentiation. Data are represented as mean ± SEM of *n* = 4 cell lines for each haplotype group. **(D)** Representative immunofluorescent images at day 30 of differentiation with neuronal markers MAP2, TAU, GABA, and BRN2 as well as the neural progenitor marker NESTIN and the glia marker GFAP. See also Supplementary Figures 1, 2. **(E)** Immunofluorescent images of axonal markers SMI312 (neurofilament) and β-3-TUBULIN (microtubules) show a growing and maturing neuronal network over the differentiation time of 30 days. **(F)** Immunofluorescent staining with the presynaptic marker SYNAPSIN and the postsynaptic marker GEPHYRIN over time show presents of pre- and postsynapses along the axons (stained with SMI312) at day 30 of differentiation. Immunofluorescent images are taken from an H2/H2 cell line.

First, we stained the neural progenitor marker NESTIN, the neuronal marker MAP2 and the glial marker GFAP at all time points (Figure 2A and Supplementary Figures 1A,B). Nearly all proliferating cells expressed NESTIN (96%) while only 16% were MAP2 positive, indicating that some spontaneous differentiation is occurring in those cells. After 5 days of differentiation, MAP2 staining was upregulated significantly to 90% and stayed on this high level until day 30. NESTIN immunoreactive cells on the other hand were continuously decreasing until only 10% at day 15 and 30. The remaining NESTIN positive cells at day 30 were MAP2 negative and displayed a non-neuronal morphology (Figure 2D). This indicated an insufficient differentiation into neurons for these few cells. Supplementary Figure 2A shows similar MAP2 staining and cell morphology at 30 days of differentiation for all cell lines. Glial cells were absent from cell culture in nearly all cell lines over the whole differentiation period (Figure 2A). Only in one cell line, GFAP positive cells could be detected at day 30 (3%; Figure 2D).

Initially described differentiation of iPSCs into neurons via *NGN2* overexpression have found expression of cortical and GABAergic markers in the resulting neurons (Zhang et al., 2013). We therefore stained the cells for the neurotransmitter GABA (Figures 2B,D and Supplementary Figure 1C) and the cortical marker BRN2 (Figures 2C,D and Supplementary Figure 2D) for further specification of the neuronal identity of our cells. In expanding smNPCs, a strong unspecific staining with the GABA antibody was visible (Supplementary Figure 1C). This time point was therefore excluded from quantitative analysis of GABAergic cells. At day 5 of differentiation, only 7% of neurons expressed GABA. But with ongoing differentiation time, GABA is significantly upregulated so that the percentage of GABAergic neurons increased significantly to 61% at 30 days of differentiation. Until day 15, the signal was mainly localized in the soma and soma-near processes (Supplementary Figure 1C), at day 30, however, GABA expression was also visible along the neuronal network (Figure 2D). Although there was some variation in the signal intensity of the GABA staining (Supplementary Figure 2C) and the percentage of GABAergic neurons between cell lines (ranging from 41 to 78% at day 30), there was no statistically significant difference between the two haplotype groups H1/H1 and H2/H2 (Figure 2B).

Next, cells were stained with cortical layer 2/3 marker BRN2 (Figure 2C). Because BRN2 lacks specificity in expanding smNPC and early differentiating cells, we assessed BRN2 staining only at day 30 of differentiation, in combination with the pan-neuronal marker TAU as normalization for all neurons (Figure 2D). At this time point, on average 65% of TAU-positive neurons expressed BRN2 in both cell line groups. Supplementary Figure 2D shows a comparable staining pattern for the cortical marker BRN2 and the neuronal marker TAU for all cell lines at day 30 of differentiation.

Furthermore, one representative cell line was stained with the axonal markers SMI312 (neurofilament) and β-3-TUBULIN (microtubules; Figure 2E). Both markers showed a growing and maturing neuronal network over the differentiation time of 30 days. The same cell line was additionally stained with the presynaptic marker SYNAPSIN and the postsynaptic marker GEPHYRIN which show the presents of pre- and postsynapses along the axons at day 30 of differentiation (Figure 2F).

In general, NESTIN and MAP2 immunostaining could show an effective and fast differentiation of *NGN2*_smNPCs into 90% neurons via *NGN2* overexpression. Although there were some minor differences, all cell lines had a comparable differentiation efficiency. Additionally, we could show the cortical and GABAergic identity of our cells at day 30 of differentiation. All in all, the cells generated a dense neuronal network and expressed important neuronal markers.

### TAU Expression in Differentiated Neurons With Homozygous H1 or H2 *MAPT* Haplotypes

Although the *MAPT* haplotype spans a region of 1.8 Mb with several genes, the *MAPT* gene coding for TAU is of special interest when investigating the underlying mechanisms of the H1-dependent risk for tauopathies. Some studies have shown a differential TAU expression between H1/H1 and H2/H2 genotypes (mainly on mRNA level; Kwok et al., 2004; Caffrey et al., 2006; Myers et al., 2007). We therefore studied the TAU expression on an mRNA as well as on a protein level in our cell lines.

**FIGURE 3:**
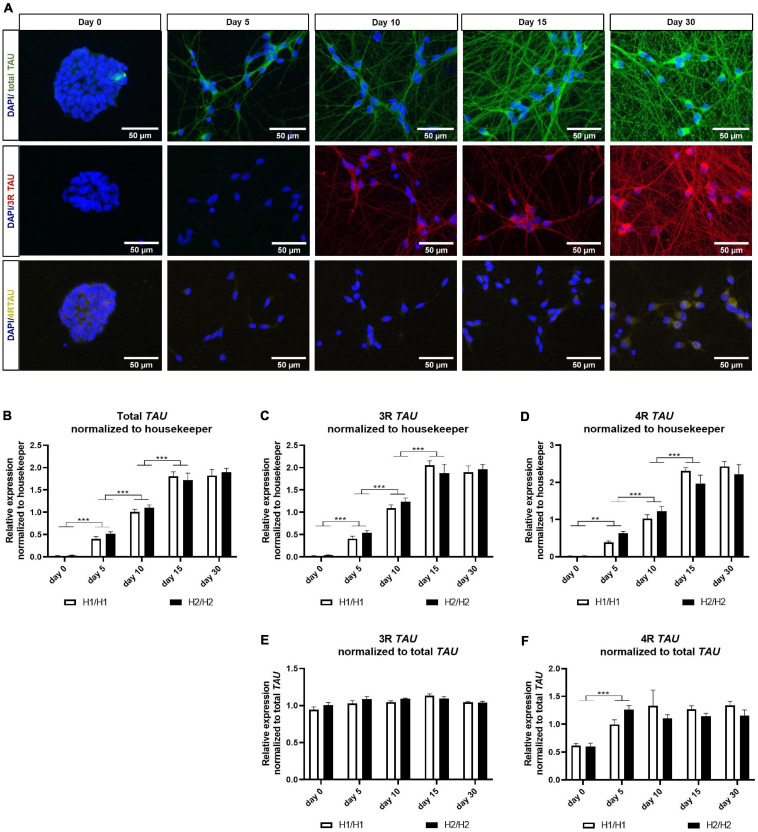
Immunofluorescent and qPCR analysis of TAU and its isoforms. **(A)** Immunofluorescent images of total TAU, 3R TAU, and 4R TAU over differentiation time of 30 days. Total TAU and 3R TAU stain the axons and the soma of cells from day 5 and 10 onward, respectively, and show a maturing neuronal network with increasing differentiation time. 4R TAU is only detectable at day 30 of differentiation. Images are taken from an H2/H2 cell line. **(B–D)** qPCR data for total *TAU* **(B)** 3R *TAU* isoform **(C)** and 4R *TAU* isoform **(D)** normalized to the three housekeeping genes UBQLN1, PSMC1, and GPBP1. Total *TAU* as well as 3R and 4R *TAU* mRNA levels increase significantly with differentiation time until day 15. No expression differences could be detected between H1/H1 and H2/H2 haplotype groups. Data are represented as mean ± SEM of *n* = 4 cell lines for each haplotype group. For each cell line at least two independent repeats were analyzed. Two-way ANOVA followed by Tukey’s test was performed. **p < 0.01, ***p < 0.001. **(E,F)** qPCR data for 3R and 4R TAU isoform normalized to total TAU levels. While 3R mRNA levels relative to total TAU levels stay constant over differentiation period, 4R TAU levels increase significantly from day 0 to day 5 and then also remain unchanged. No expression differences could be detected between H1/H1 and H2/H2 haplotype groups. Data are represented as mean ± SEM of *n* = 4 cell lines for each haplotype group. For each cell line at least two independent repeats were analyzed. Two-way ANOVA followed by Tukey’s test was performed. **p < 0.01, ***p < 0.001.

First, TAU expression was visualized during the differentiation period of 30 days by staining one cell line with total TAU, 3R TAU, and 4R TAU antibodies (Figure 3A). Total TAU and 3R TAU displayed a similar staining pattern with signals in the soma as well as in the axons of neurons. A staining was visible from day 5 (total TAU) or day 10 (3R TAU) onward and showed a more extensive neuronal network over time. The 4R TAU isoform was only detectable at a differentiation time of 30 days via immunostaining and even then, the signal remained weak and mainly visible in the soma and only faintly in the axons. At day 30 of differentiation all cell lines were stained with the total TAU antibody (Supplementary Figure 2B). Although there were a few minor differences in TAU staining, the neuronal network looks overall very similar and there were no obvious differences visible that manifested between the *MAPT* haplotype groups.

To investigate *TAU* expression on an mRNA level, we performed qPCR with primers specific for total *TAU*, 3R *TAU*, or 4R *TAU* isoforms (Figures 3B–F). We could observe a significant increase in all three mRNA levels from day 0 to day 15 of differentiation (Figures 3B–D). From day 15 to day 30, mRNA levels remained unchanged for both isoforms and total *TAU*. When normalizing the 3R and 4R data to total *TAU* levels (Figures 3E,F), the 3R *TAU* level stayed unchanged over time. 4R *TAU* on the other hand increased significantly from day 0 to day 5, indicating an upregulation of this isoform with the start of differentiation. For the remaining differentiation time the 4R level stagnated. No expression differences for total *TAU* or any of the isoforms could be detected between H1/H1 and H2/H2 haplotype groups.

**FIGURE 4:**
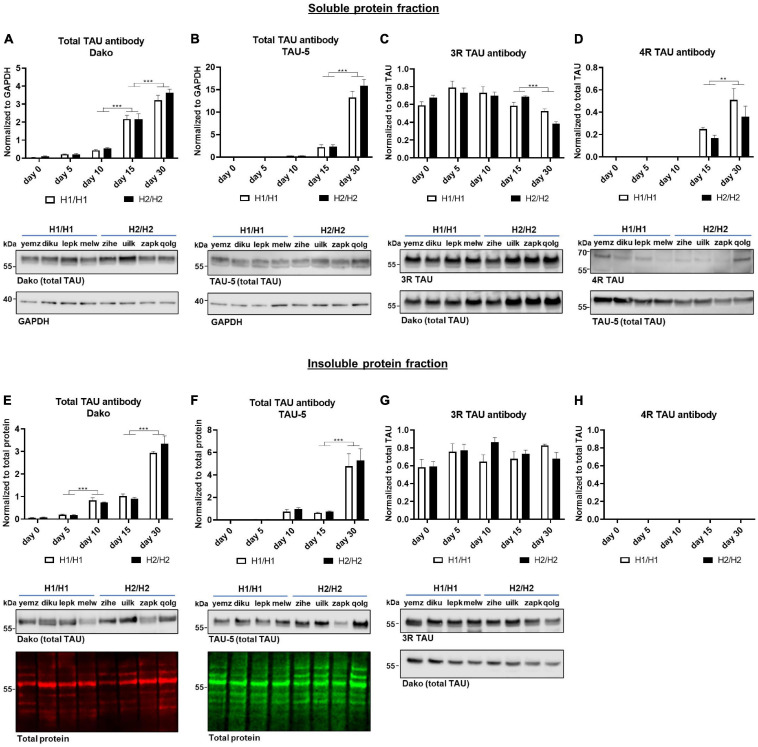
Western blot analysis over time with total TAU, 3R, and 4R TAU antibodies. **(A–D)** Western blot of soluble protein fraction. Total TAU expression was assessed with Dako and TAU-5 antibody **(A,B)** and normalized to GAPDH. Total TAU levels increase significantly over time. While 3R TAU expression relative to total TAU **(C)** stays constant until day 15 and then decreases significantly, 4R TAU **(D)** is only detectable from day 15 onward and increases further to day 30. **(E–H)** Western blot of insoluble protein fraction. Total TAU levels were assessed with Dako and TAU-5 antibody **(E,F)** and normalized to total protein. Total TAU expression in insoluble fraction increased significantly over time. 4R TAU **(H)** is not detectable while the 3R TAU level **(G)** remains unchanged over time when normalized to total TAU levels. No significant differences could be detected between H1/H1 and H2/H2 haplotype groups for any of the antibodies or fractions. Data are represented as mean ± SEM of *n* = 4 cell lines for each haplotype group. For each cell line at least three independent repeats were analyzed. Two-way ANOVA followed by Tukey’s test was performed. **p < 0.01, ***p < 0.001. Displayed Western blot pictures are representative blots from day 30 of differentiation for the respective antibody.

**FIGURE 5:**
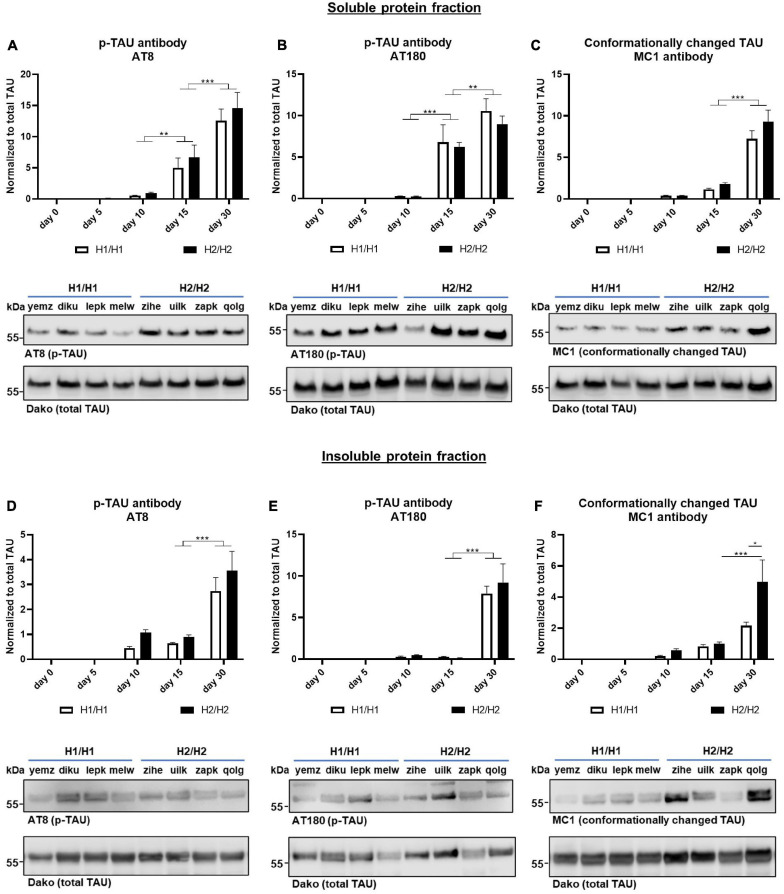
Western blots for phosphorylated and conformationally changed TAU. Phosphorylated TAU (p-TAU) levels were investigated with the antibodies AT8 and AT180, conformationally altered TAU is detected with the MC1 antibody. Data for all antibodies were normalized to total TAU levels. **(A–C)** In the soluble protein fraction, p-TAU **(A,B)** and MC1 positive TAU **(C)** levels increase significantly over time with day 30 displaying the highest levels. **(D–F)** In the insoluble protein fraction, p-TAU **(D,E)** and MC1 positive TAU **(F)** levels also increase significantly over time. No significant differences could be detected between H1/H1 and H2/H2 haplotype groups for any of the antibodies except for MC1. Here, at day 30, the level in the H2/H2 group is significantly higher in the insoluble fraction than in the H1/H1 group. Data are represented as mean ± SEM of *n* = 4 cell lines for each haplotype group. For each cell line at least three independent repeats were analyzed. Two-way ANOVA followed by Tukey’s or Sidak’s test (MC1 Western blot) was performed. *p < 0.05, **p < 0.01, *** *p* < 0.001. Displayed Western blot pictures are representative blots from day 30 of differentiation for the respective antibody.

Next, we studied the expression of the TAU protein with multiple TAU antibodies at different time points in the soluble as well as in the insoluble protein fraction (Figures 4, 5). The total TAU levels were assessed with two different antibodies: the polyclonal TAU antibody from Dako and the monoclonal TAU-5 antibody (Figures 4A,B). Both antibodies showed a strong increase in TAU expression during differentiation with the highest TAU levels at day 30. The same was visible in the insoluble fraction (Figures 4E,F). For 3R and 4R TAU, the data was normalized to total TAU to assess changes in isoform levels independent from overall TAU levels. 4R TAU was only detectable from day 15 onward and with a very weak signal, making 3R TAU the predominant TAU isoform expressed in this cell model during early maturation stages (Figures 4C,D). The 3R TAU level stayed constant during the differentiation time until day 15 and then significantly decreased to day 30. In accordance with these data, the 4R TAU level increased significantly from day 15 to day 30. In the insoluble protein fraction, no 4R TAU could be detected and the 3R TAU level remained unchanged over the whole differentiation period (Figures 4G,H). There were no significant expression differences in total TAU nor in one of the TAU isoforms between cell lines of different *MAPT* haplotype groups, neither in the soluble nor in the insoluble fraction.

To assess the levels of phosphorylated TAU (p-TAU), two different p-TAU antibodies were used that bind to different phosphorylated epitopes on the TAU protein. The AT8 antibody binds to phosphorylated Serin202 and Threonin205 while the AT180 clone binds to Threonin230. The p-TAU signal was normalized to the total TAU signal. Both antibodies could detect p-TAU from day 5 onward (Figures 5A,B). With further differentiation time, the p-TAU levels increased greatly until day 30, indicating that with extended differentiation time, more TAU becomes phosphorylated. Also in the insoluble fraction, we saw a significant rise of p-TAU until day 30 (Figures 5D,E). For both fractions and antibodies, p-TAU levels did not differ significantly, when comparing the H1/H1 and H2/H2 groups at any time point.

Additionally, we studied the levels of conformationally changed TAU with the MC1 antibody that recognizes a disease-specific TAU conformation dependent on the N-terminus and a sequence in the third microtubule binding repeat (Jicha et al., 1997; Figures 5C,F). Again, the data was normalized to total TAU levels. As already shown for the p-TAU antibodies, the MC1 signal increased during the differentiation time in the soluble as well as in the insoluble protein fraction with the highest levels of MC1 positive TAU at day 30. While there was no difference in conformationally changed TAU levels at any time point in the soluble fraction between *MAPT* haplotype groups, in the insoluble fraction at day 30 of differentiation, the H2/H2 cell lines showed significantly more MC1 positive TAU then the H1/H1 group. Although it needs to be noted that within the H2/H2 group there was great variability between cell lines, indicated by the great SEM and visible in the Western blots.

Since some studies have reported that only sub-haplotypes of the H1 haplotype are associated with a higher risk for neurodegenerative disease (Myers et al., 2005; Ezquerra et al., 2011), we genotyped the H1/H1 cell lines according to the SNPs that define the H1 sub-haplotypes (Table 2; Pittman et al., 2005). However, none of the H1 cell lines was homozygous for a sub-haplotype (Table 3), therefore, no further investigation was carried out.

**TABLE 3:** Genotyping of H1/H1 cell lines for sub-haplotypes.

Cell line	rs1467967	rs242557	rs3785883	rs2471738	rs8070723	rs7521
yemz	A/A	A/G	G/A	T/C	A/A	A/A
diku	A/A	A/A	G/G	C/T	A/A	G/G
lepk	A/A	A/A	G/G	T/C	A/A	G/A
melw	A/A	A/G	G/G	C/T	A/A	A/A

In summary, we could show that our cell model started to express the 4R TAU isoform on a protein level after 15 days of differentiation (even earlier on an mRNA level), although 3R TAU protein remained predominant until day 30. We could not find any haplotype specific differences in total TAU, 3R TAU, or 4R TAU expression nor in the phosphorylation levels neither in the soluble or in the insoluble protein fraction between cell lines of the two different *MAPT* genotype groups. Interestingly, our data showed a higher level of conformationally altered TAU in the insoluble protein fraction of H2/H2 cells compared to H1/H1.

### α-SYNUCLEIN Expression in Differentiated Neurons With Homozygous H1 or H2 *MAPT* Haplotypes

The H1 haplotype is not only a major risk factor for several tauopathies but is also associated with a higher probability to develop PD (Zabetian et al., 2007; Ezquerra et al., 2011; Di Battista et al., 2014). This is somehow surprising since the primarily affected protein in PD is α-SYNUCLEIN and not TAU. Therefore, after studying TAU expression in our cell model, we also investigated the expression levels of α-SYNUCLEIN.

**FIGURE 6:**
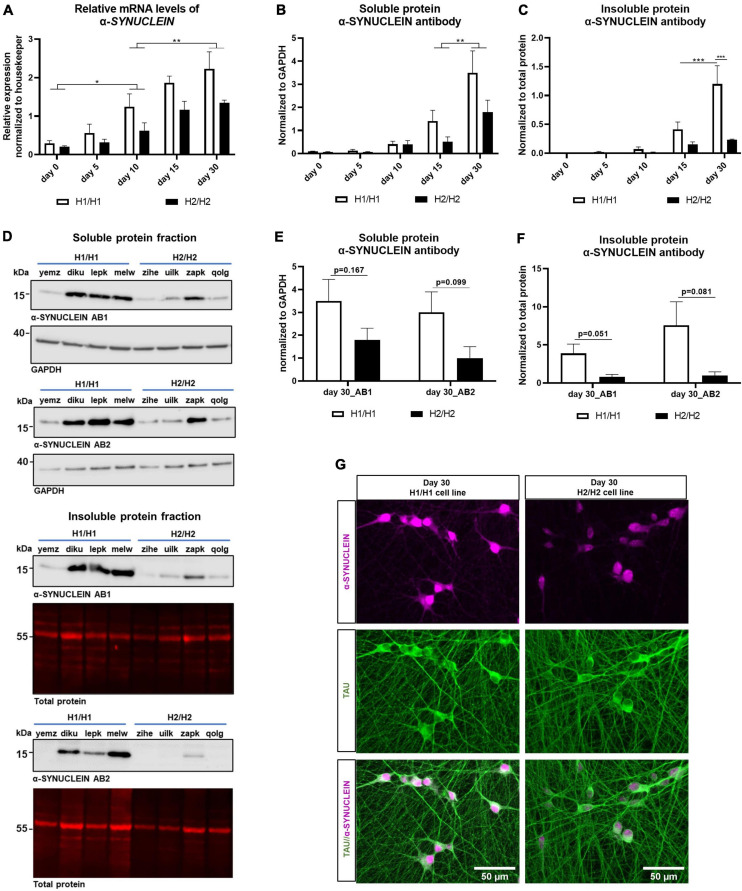
Analysis of α-SYNUCLEIN expression over time on protein and mRNA level. **(A)** qPCR for α*-SYNUCLEIN* over differentiation time of 30 days. α*-SYNUCLEIN* levels increase significantly over time. Data are represented as mean ± SEM of *n* = 4 cell lines for each haplotype group. For each cell line at least two independent repeats were analyzed. Two-way ANOVA was performed and revealed a significantly higher expression in H1/H1 cells (*p* = 0.0011). For the factor time, a Tukey’s test was performed. *p < 0.05, **p < 0.01. **(B,C)** Western blot of soluble and insoluble protein fraction with α-SYNUCLEIN antibody over differentiation time of 30 days. α-SYNUCLEIN levels increase significantly over time in both fractions. Data are represented as mean ± SEM of *n* = 4 cell lines for each haplotype group. For each cell line at least three independent repeats were analyzed. Two-way ANOVA was performed followed by Tukey’s or Sidak’s test and revealed an overall significantly higher expression in H1/H1 cells (*p* = 0.036) compared to H2/H2 in the soluble fraction and a significantly higher level in H1/H1 cells at day 30 in the insoluble fraction. **p < 0.01, ***p < 0.001. **(D)** Representative Western blots from 30-day old cells for two α-SYNUCLEIN antibodies (AB1 and AB2) for the soluble and the insoluble fraction. **(E,F)** Western blot of soluble and insoluble protein with two α-SYNUCLEIN antibodies (AB1 and AB2) at day 30 of differentiation could not detect a significant difference between H1/H1 and H2/H2 groups with unpaired *t*-test. Data are represented as mean ± SEM of *n* = 4 cell lines for each haplotype group. For each cell line at least three independent repeats were analyzed. **(G)** Immunofluorescent staining of two representative cell lines for α-SYNUCLEIN and TAU at day.

First, mRNA levels were measured by qPCR (Figure 6A). α*-SYNUCLEIN* mRNA levels increased continuously over the differentiation time of 30 days. Interestingly, cell lines with the H1/H1 haplotype expressed significantly more α*-SYNUCLEIN* than H2/H2 cell lines (two-way ANOVA *p* = 0.011).

Next, the α-SYNUCLEIN protein expression was assessed by western blot in the soluble as well as in the insoluble protein fraction. Like the mRNA level, expression of α-SYNUCLEIN protein increased during the differentiation time in the soluble and in the insoluble fraction continuously until 30 days (Figures 6B,C). In accordance with the mRNA results, also on the protein level (soluble fraction) the H1/H1 haplotype group expressed significantly more α-SYNUCLEIN than the H2/H2 group (two-way ANOVA, *p* = 0.036). In the insoluble fraction, there was a statistically significant difference between *MAPT* groups only at day 30 of differentiation although the trend was also visible at earlier time points.

To further verify the western blot result, samples from day 30 of both fractions (time point with highest α-SYNUCLEIN level) were again analyzed with a different α-SYNUCLEIN antibody (Figures 6D–F). In Figures 6E,F the results for day 30 with both antibodies are displayed. The second α-SYNUCLEIN antibody could replicate the results from the originally used antibody for both fractions (see also Figure 6D). Statistical testing with an unpaired *t*-test, however, could not detect a statistically significant difference between H1/H1 and H2/H2 cell lines for neither antibody in both fractions at this time point.

Two representative cell lines, one from each *MAPT* haplotype group, was chosen to visualize the differential α-SYNUCLEIN protein expression with immunocytochemistry at day 30 of differentiation (Figure 6G). See also Supplementary Figure 3 for high-magnification images auf TAU and α-SYNUCLEIN staining showing no clear colocalization of the two proteins.

Several SNPs in the *SNCA* gene coding for α-SYNUCLEIN have been reported to increase the risk for synuleinopathies and potentially influence the expression of α-SYNUCLEIN (Campêlo and Silva, 2017; see Table 4). To investigate whether the differential expression in H1 versus H2 cells could be explained by SNPs in the *SNCA* gene, we selected SNPs that have reportedly been associated with increased risk for PD and/or with higher expression of α-SYNUCLEIN and genotyped our cell lines accordingly (Table 5).

**TABLE 4:** Single nucleotide polymorphisms that have reportedly been associated with increased risk for Parkinson’s disease (PD) and/or with higher expression of α-SYNUCLEIN.

5′ UTR (promotor region)	Intron 4	3′ UTR
rs2619363 (Winkler et al., 2007)	rs2736990 (McCarthy et al., 2011; Pan et al., 2013)	rs356165 (Winkler et al., 2007; Cardo et al., 2012)
rs2619364 (Winkler et al., 2007)		rs356219 (Linnertz et al., 2009; Mata et al., 2010)
rs2583988 (Winkler et al., 2007)		rs181489 (Elbaz et al., 2011)
Rep1 (Chiba-Falek and Nussbaum, 2001; Fuchs et al., 2008; Cronin et al., 2009)		rs356182 (Cheng et al., 2016)

**TABLE 5:** Genotype of cell lines for certain SNPs in the *SNCA* gene.

Cell line	rs2583988	rs2610364	rs2619363	rs356182	rs181489	rs356219	rs356165	rs2736990
yemz	C/T	A/G	G/T	G/A	T/C	G/A	G/A	G/A
diku	C/T	A/G	G/T	G/G	T/T	G/G	G/G	G/G
lepk	T/T	G/G	T/T	G/G	T/T	G/G	G/G	G/G
melw	C/T	A/G	G/T	G/A	T/C	G/A	G/A	G/A
zihe	C/C	A/A	G/G	G/A	T/C	G/A	G/A	G/A
uilk	C/C	A/A	G/G	A/A	C/C	A/A	A/A	A/A
zapk	C/C	A/A	G/G	A/A	C/C	A/A	A/A	A/A
qolg	C/C	A/A	G/G	A/A	C/C	A/A	A/A	A/A

Additionally to SNPs, a complex, polymorphic microsatellite called Rep1 (D4S3481) located approximately 10 kb upstream of the *SNCA* transcription start site has been linked to an increased risk for PD (Campêlo and Silva, 2017). The number of dinucleotide repeats in the microsatellite sequence can differ between individuals and studies have shown that longer repeats of Rep1 are associated with increased transcription levels of α*-SYNUCLEIN* (Chiba-Falek and Nussbaum, 2001; Cronin et al., 2009). Therefore, we also genotyped the cell lines according to their repeat sequence of Rep1 (Table 6). When grouping the cell lines according to their SNP or Rep1 genotype, no pattern that would explain the α-SYNUCLEIN expression data could be identified.

**TABLE 6:** Sequence and genotype of cell lines for Rep1 microsatellite^a^ near the *SNCA* gene.

Cell line→ genotype^b^	TC	TT	TC	TA	CA	Genotype according to length
Reference sequence→ 0/0	10	1	10	8	10	259/259
yemz→ 0/1	11	1	9	10	10	259/263
diku→ 1/1	10/11	1	10	8	11/10	261/261
lepk→ 0/1	11	1	11	7	10	259/261
melw→ 0/1	11	1	11	7	10	259/261
zihe→ 0/0	10	1	10	8	10	259/259
uilk→ 1/1	10	1	10	8	11	261/261
zapk→ 0/1	10	1	11	7	11	259/261
qolg→ 0/1	10	1	10	8	11	259/261

*^*a*^Microsatellite Rep1 consists of dinucleotide combination that can differ in number of repeats and thus in length (TC)_*x*_(TT)(TC)_*y*_(TA)_*z*_(CA)_*w*_ → PCR products run at length of 259 bp, 261 pb, and 263 pd. The longer versions 261 bp and 263 bp have been associated with risk for PD and increased α-SYNUCLEIN expression.*

*^*b*^When sequence is different to reference sequence only on one allele, only this deviant sequence is listed in the table.*

In summary, we detected a significantly higher α-SYNUCLEIN level in cell lines with the H1 haplotype compared to cells with the H2 haplotype. This difference manifested on an mRNA as well as on a protein level over the whole differentiation period. Variations in the α*-SYNUCLEIN* gene causing this expression pattern could not be identified.

### Toxin Treatment of Differentiated Neurons With Homozygous H1 or H2 *MAPT* Haplotypes

Although there are several genetic risk factors that increase the possibility for developing a neurodegenerative disease (such as the *MAPT* haplotype), they do most likely not act alone but rather in concert with other genetic variations and/or environmental factors. Therefore, we investigated whether cell lines with homozygous H1 or H2 *MAPT* genotypes would differ in their vulnerability to external stressors like toxins.

**FIGURE 7:**
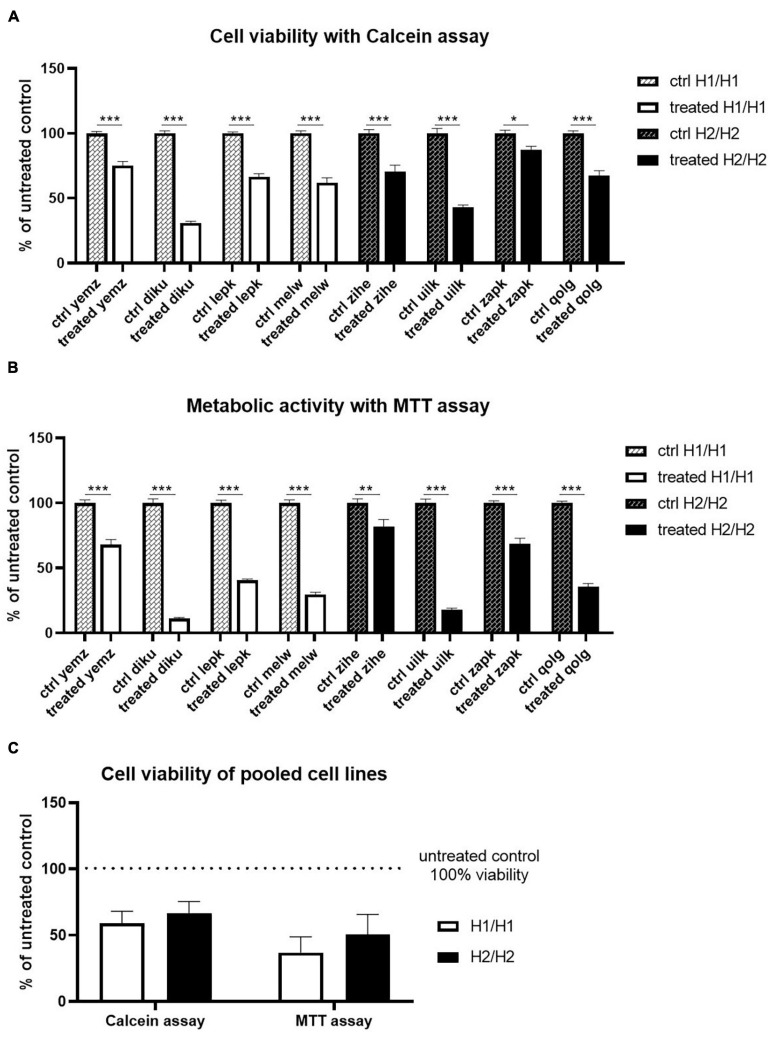
Treatment of cells with Annonacin. **(A,B)** Cell viability was analyzed using Calcein and MTT assay, respectively. Annonacin treatment reduced cell viability significantly in all cell lines. Data are represented as mean ± SEM of *n* = 8 independent experiments per cell line. Two-way ANOVA followed by Sidak’s test was performed. *p < 0.05, **p < 0.01, ***p < 0.001 **(C)** Pooling of cell lines into haplotype groups does not detect any significant difference in cell viability between H1/H1 and H2/H2 groups after Annonacin treatment. Data are represented as mean ± SEM of *n* = 4 cell lines for each haplotype group. Unpaired *t*-test was performed.

We treated the cells with the mitochondrial inhibitor Annonacin which has been reported to cause atypical parkinsonism (Caparros-Lefebvre and Elbaz, 1999; Höglinger et al., 2005). 26-day old cells were treated with 800 nM Annonacin for 4 days to cause a significant toxic effect in all cell lines. At day 30 of differentiation, cell viability was assessed with Calcein staining, while mitochondrial activity was measured with an MTT assay. All cell lines showed a significant reduction of cell viability and metabolic activity 4 days after treatment. The toxic effect of Annonacin, however, differed greatly between the cell lines independent of the haplotype (Figures 7A,B). Reduced viability with Calcein staining ranged from 80 to 30% and mitochondrial activity from 80 to 10%. Strong reduction of mitochondrial function co-occured with greater reduction in general viability. Although there are variations between the cell lines in general, when comparing the two *MAPT* haplotype groups, there were no significant differences in the toxic effect of Annonacin neither in viability measured with Calcein nor the metabolic activity measured by the MTT assay (Figure 7C).

All in all, we could not detect a *MAPT* haplotype-dependent differential vulnerability to the toxin Annonacin.

### TAU Uptake in Differentiated Neurons With Homozygous H1 or H2 *MAPT* Haplotypes

**FIGURE 8:**
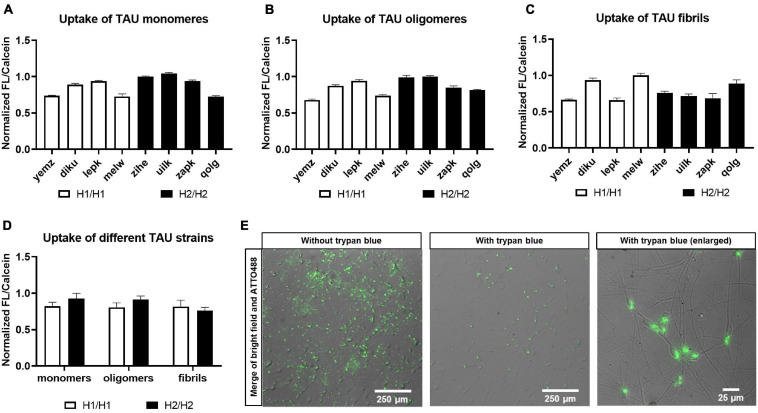
Treatment of cells with TAU seeds. Cells were treated with recombinant and fluorescently labeled TAU and uptake was monitored. Cells were treated with either TAU monomers **(A)**, TAU oligomers **(B)**, or fibrils **(C)**. Data are represented as mean ± SEM of *n* = 3 technical repeats per cell line. **(D)** Pooling of cell lines into haplotype groups does not detect any significant difference in TAU uptake between H1/H1 and H2/H2 groups. Data are represented as mean ± SEM of *n* = 4 cell lines for each haplotype group. Unpaired *t*-test was performed. **(E)** Merge of bright field and fluorescent images of cells after treatment with ATTO488-labeled TAU seeds before and after addition of trypan blue for quenching of the outside signal.

For the spreading of pathology throughout different brain regions in neurodegenerative tauopathies, TAU is released from diseased cells and taken up by neighboring cells in a Prion-like manner. Therefore, we tested in our cell model the ability of various cell lines to take up TAU monomers and aggregates generated from recombinant protein. We prepared fluorescently labeled TAU strains including monomers, oligomers, and fibrils (see Supplementary Figure 4 for characterization of aggregates). The cells were treated with each type of TAU strain and intracellular fluorescence signals were measured by a plate reader, while the extracellular signal was quenched with trypan blue (Figure 8). Fluorescence signal from TAU uptake was normalized to the whole cell stained with Calcein to eliminate the effect of variation in cell seeding for each line (see Supplementary Figure 5 for effect of trypan blue on plate reader signal and toxicity assessment of TAU treatment). Although there were some differences in the intensity of internalized TAU signal between the cell lines, we could not detect any *MAPT* haplotype-dependent differences in uptake signal for different TAU strains (Figure 8D).

## Discussion

Over the last years, several protocols have been established to differentiate iPS cells into neurons to model neurodegenerative diseases. Direct differentiation methods include differentiation times from 70 to 200 days or longer (Iovino et al., 2015; Sposito et al., 2015; Silva et al., 2016; Garcia-Leon et al., 2018) and can vary greatly in the obtained cell type composition (Volpato et al., 2018). When comparing several cell lines generated from individuals with a different genetic background, reducing the variability within the differentiation process is of great importance. We therefore first converted the iPSC into smNPC and then used *NGN2* overexpression for a fast and efficient differentiation into a high percentage (90%) of neurons that express important neuronal markers (Dhingra et al., 2020). All cell lines showed a comparable neuronal morphology when stained with MAP2 and TAU. Glial cells are either not present or only to a very small percentage at day 30. Cell lines of both haplotype groups showed similar differentiation efficiency and comparable percentages of cortical and GABAergic neurons. We could therefore show that our cell model is robust and suitable to compare different cell lines. The easily expandable *NGN2*_smNPCs and the short differentiation time makes the model additionally applicable for high-throughput approaches.

One of the main challenges when using iPSC to model tauopathies is the expression of the adult 4R TAU isoform. Differentiated neurons from iPSC mostly remain in an immature state of development (Miguel et al., 2019). Therefore, the predominantly expressed TAU isoform is the fetal 0N3R isoform (Fong et al., 2013; Nakamura et al., 2019). With most differentiation protocols, only extensive differentiation times (at least 100 days) are able to induce the developmental switch leading to the expression of 4R TAU (Iovino et al., 2015; Sposito et al., 2015; Garcia-Leon et al., 2018). Although, the predominant TAU isoform is also the 0N3R in our model, we could show that with our protocol 4R TAU is already detectable on a protein level at day 15 of differentiation and increases further until day 30. This indicates that the *NGN2* overexpression does not only very efficiently induce a neuronal phenotype but also speeds up the maturation process, making our model a valuable tool to investigate TAU-related disease.

Furthermore, total TAU expression steadily increases during differentiation and in parallel also the TAU levels in the insoluble protein fraction increases, indicating that more TAU is aggregating with aging of the neurons. Additionally, TAU becomes more and more phosphorylated with ongoing differentiation time.

Since the *MAPT* haplotype has been reported a major risk factor for several neurodegenerative diseases, several studies have tried to understand the underlying mechanism, with *MAPT* being the predominant gene of interest. Experiments in neuroblastoma cell lines have reported a higher transcription efficiency of the H1 promotor over the H2 promotor (Kwok et al., 2004). In human brain, differential expression on an mRNA level was observed, although those findings have been contradictory between different studies. de Jong et al. (2012) and Valenca et al. (2016) for example reported higher *MAPT* expression in H1 haplotype brains compared to H2. Caffrey et al. (2006) and Trabzuni et al. (2012) on the other hand could not detect an overall higher *MAPT* expression from H1 alleles. Additionally, Myers et al. (2007) only found an increased *MAPT* expression in brains homozygous for the H1 sub-haplotype H1c compared to all other haplotypes. Some studies have also reported higher levels of transcripts that include exon 10 (4R TAU isoforms; Caffrey et al., 2006; Myers et al., 2007; Majounie et al., 2013). The only study so far conducted with iPSC concentrated on the allele-specific expression of *MAPT* transcripts in H1/H2 heterozygous cells (Beevers et al., 2017) reporting a higher overall TAU transcript from H1. A difference in exon 10 + transcripts between the alleles could not be found.

Since all the described studies focused on the expression levels of mRNA, it remains unclear if and how differences in the transcript levels would translate into differences on the protein level. Our study is therefore the first systematic comparison of several iPSC lines that are homozygous for either the H1 or the H2 haplotype investigating the *MAPT* expression not only on an mRNA but also on the protein level.

In accordance with the studies in iPSC from Beevers et al. we could not detect differences in the mRNA expression levels of the 4R TAU isoform between the two haplotype groups. However, also in the total TAU transcripts, we could not detect a difference between H1 and H2 cell lines. The same holds true for the 3R TAU isoform. Also on the protein level, there is no statistical difference between the haplotype groups, although the sample size of four cell lines per haplotypes may limit the identification of inter-haplotype differences when intra-individual variance is predominant.

Surprisingly, when we analyzed the levels of conformationally changed TAU, we could observe significantly higher levels in the insoluble fraction of H2/H2 cell lines when differentiating for 30 days. The variability between cell lines is quite high, especially within the H2/H2 group, but the detected difference is significant. A study conducted in patients with AD and Lewy body disease (LBD) compared TAU deposition in H1 and H2 carriers (Wider et al., 2012). Although AD and LBD are not primary tauopathies they can still display secondary tau pathology like neurofibrillary tangles (NFT; Jellinger, 2011). Wider et al. connected a higher NFT burden with the H2 haplotype. This finding would support our observation of higher levels of conformationally changed TAU in the insoluble protein fraction in H2 cell lines which could point to a higher aggregation in this haplotype.

Since the H1 haplotype is not only a major risk factor for tauopathies but also significantly increases the risk for PD, we also investigated the α-SYNUCLEIN levels in our cells. Interestingly, we could find significantly higher expression levels in H1/H1 cell lines compared to H2/H2 cell lines on the mRNA as well as on the protein level. How the H1 haplotype could influence the risk for synucleinopathies remains unclear. As mentioned above, TAU pathology can occur in synucleinopathies such as PD or Dementia with Lewy bodies (DLB). Two studies support our findings of higher α-SYNUCLEIN expression between H1 and H2 carriers on a pathophysiological level (Colom-Cadena et al., 2013; Robakis et al., 2016). α-SYNUCLEIN pathology, like the Lewy body (LB) count, in brains of patients with DLB or PD was significantly higher in carriers of the H1/H1 genotype. The TAU pathology, like NFT, however, did not significantly differ between H1 and H2 case. The Wider et al. (2012) study also found a higher LB count in patients with AD and DLB that carried the H1 haplotype in addition to a higher NFT count in H2/H2 carriers. This study supports both findings in our cell model: higher α-SYNUCLEIN levels in H1/H1 cells and higher conformationally changed TAU levels in the insoluble fraction in H2/H2 cells.

How the H1 haplotype could influence the increased expression of α-SYNUCLEIN already on an mRNA level is not clear. Since expression levels of α-SYNUCLEIN vary also within the haplotype groups, differential expression could be influenced by other genetic factors independent from the *MAPT* haplotype. We therefore genotyped our cell lines for genetic variants in the *SNCA* gene but could not identify an association with any of the chosen variants and the described α-SYNUCLEIN expression pattern.

Since the *MAPT* haplotype does not only comprise the *MAPT* gene but several other genes, the possibility that one of those genes could be differentially regulated in the H1 haplotype and then in turn regulate the α*-SYNUCLEIN* expression cannot be excluded. So far, little is known about the genes located in the *MAPT* haplotype. de Jong et al. (2012) reported that the inversion polymorphism of H2 affects expression of multiple other genes next to *MAPT*, none of which is known to regulate transcription of the *SNCA* gene.

In conclusion, we can present a valuable cell resource of 8 iPSC lines selected according to their *MAPT* haplotype, that we converted into *NGN2*_smNPC. Their fast and efficient differentiation into neurons makes them suitable for high-throughput approaches. Although limited with a sample size of four H1/H1 and four H2/H2 cell lines, this resource creates a physiologically relevant tool to investigate genetic variants associated with sporadic neurodegenerative diseases. We could, for example, show that H2 cell lines have higher levels of conformationally changed and thus aggregated TAU compared to H1 cells. Furthermore, we identified a differential expression of α-SYNUCLEIN between the haplotype groups. These results show that our cell resource is a valuable tool for future research with the aim to identify neurodegenerative disease mechanisms.

## Data Availability Statement

The datasets presented in this study can be found in online repositories. The names of the repository/repositories and accession number(s) can be found in the article/Supplementary Material.

## Author Contributions

TS, SS, and GH conducted the project design. TS carried out the experiments, analyzed the data, drafted the manuscript, and conceptualized the figures. AM-T carried out the TAU uptake experiments and their analysis, wrote sections of the manuscript. AD trained TS for iPSC and smNPC culture methods. ES, TS, and AD converted iPSC to NGN2_smNPC. GH, SS, PH, and WW provided conceptual guidance of the project. DT analyzed genomic data for cell line identification. FW and GH revised it critically for important intellectual content. All authors corrected and approved the final manuscript.

## Conflict of Interest

The authors declare that the research was conducted in the absence of any commercial or financial relationships that could be construed as a potential conflict of interest.

## Publisher’s Note

All claims expressed in this article are solely those of the authors and do not necessarily represent those of their affiliated organizations, or those of the publisher, the editors and the reviewers. Any product that may be evaluated in this article, or claim that may be made by its manufacturer, is not guaranteed or endorsed by the publisher.

## Footnotes

1
https://www.phe-culturecollections.org.uk/products/celllines/ipsc/search.jsp

2
https://www.hipsci.org/lines

3
http://genome-euro.ucsc.edu/index.html

4
www.graphpad.com
