# Supplementary material for: iPS Cell-Based Model for MAPT Haplotype as a Risk Factor for Human Tauopathies Identifies No Major Differences in TAU Expression
Source: Front Cell Dev Biol. 2021 Aug 31;9:726866. doi: 10.3389/fcell.2021.726866 (PMC8438159; doi:10.3389/fcell.2021.726866)
Supplement: Supplementary file 1 [file Data_Sheet_1.docx]

Supplementary Material

# Supplementary Tables

**Supplementary Table 1**: Antibodies used for immunocytochemistry

| **Antibody** | **Clone** | **Species** | **Dilution** | **Source** |
| --- | --- | --- | --- | --- |
| MAP2 | Clone HM-2 | Mouse | 1:500 | Abcam (ab11267) |
| MAP2ab | AP20 | Mouse | 1:500 | Merck (MAB378) |
| NESTIN | Polyclonal | Rabbit | 1:500 | Abcam (ab82375) |
| GFAP | 2.2B10 | Rat | 1:1000 | Life Tech (130300) |
| GABA | Polyclonal | Rabbit | 1:1000 | Sigma-Aldrich (A2052) |
| SMI-312 | SMI-312 | Mouse | 1:1000 | Abcam (ab24574) |
| SYNAPSIN | D12G5 | Rabbit | 1:500 | Cell Signaling (5297S) |
| GEPHYRIN | Polyclonal | Rabbit | 1:1000 | Abcam ab32206 |
| Total TAU | TAU-12 | Mouse | 1:500 | Merck (MAB2241) |
| 3R TAU (RD3) | Clone 8E6/C11 | Mouse | 1:500 | Millipore (05-803) |
| 4R-TAU (RD4) | Clone 1E1/A6 | Mouse | 1:100 | Millipore (05-804) |
| α‑SYNCLEIN | 14H2L1 | Rabbit | 1:500 | Invitrogen (701085) |
| β-3-TUBULIN | D71G9 | Rabbit | 1:500 | Cell Signaling (5568S) |

**Supplementary Table 2**: Primers used for qPCR

| **Target** | **Forward primer** | **Reverse primer** |
| --- | --- | --- |
| *PMSC1* | CACACTCAGTGCCGGTTAAAA | GTAGACACGATGGCATGATTGT |
| *UBQLN1* | TGCAGGTCTGAGTAGCTTGG | AACTGTCTCATCAGGTCAGGAT |
| *GPBP1* | ATCATTCGGTCTTCAACCTTCC | ATCCTCAGTTAAGGGAGCACA |
| Total *TAU* | CGTCCCTGGCGGAGGAAATA | CCCGTGGTCTGTCTTGGCTT |
| 3R *TAU* | AGGCGGGAAGGTGCAAATAG | CCTGGCCACCTCCTGGTTTATG |
| 4R *TAU* | GCCCATGCCAGACCTGAAGA | CCTCCCGGGACGTGTTTGAT |
| *α‑SYNUCLEIN* | AAGAGGGTGTTCTCTATGTAGGC | GCTCCTCCAACATTTGTCACTT |

**Supplementary Table 3**: Antibodies used for Western blot

| **Antibody** | **Clone** | **Species** | **Dilution** | **Source** |
| --- | --- | --- | --- | --- |
| Total TAU | Polyclonal | Rabbit | 1:3000 | Agilent Dako (CAC-TIP-4RT-P01) |
| Total TAU | Clone TAU-5 | Mouse | 1:1000 | Millipore (MAB361) |
| 3R TAU (RD3) | Clone 8E6/C11 | Mouse | 1:1000 | Millipore (05-803) |
| 4R TAU (RD4) | clone 1E1/A6 | Mouse | 1:300 | Millipore (05-804) |
| Phospho-TAU (Ser202, Thr205) | Clone AT8 | Mouse | 1:1000 | Life Tech (MN1020) |
| Phosphor-TAU (Thr231) | Clone AT180 | Mouse | 1:1000 | Life Tech (MN1040) |
| MC1 |  | Mouse | 1:250 | Gift from Peter Davies |
| α‑SYNUCLEIN (AB1) | polyclonal | Rabbit | 1:500 | Cell signaling (2642S) |
| α‑SYNUCLEIN (AB2) | Syn211 | Mouse | 1:500 | Invitrogen (MA5-12272) |
| β-3-TUBULIN | D71G9 | Rabbit | 1:500 | Cell signaling (5568S) |
| GAPDH | 6C5 | Mouse | 1:200 | Santa Cruz (sc-32233) |

.

# Supplementary Figures


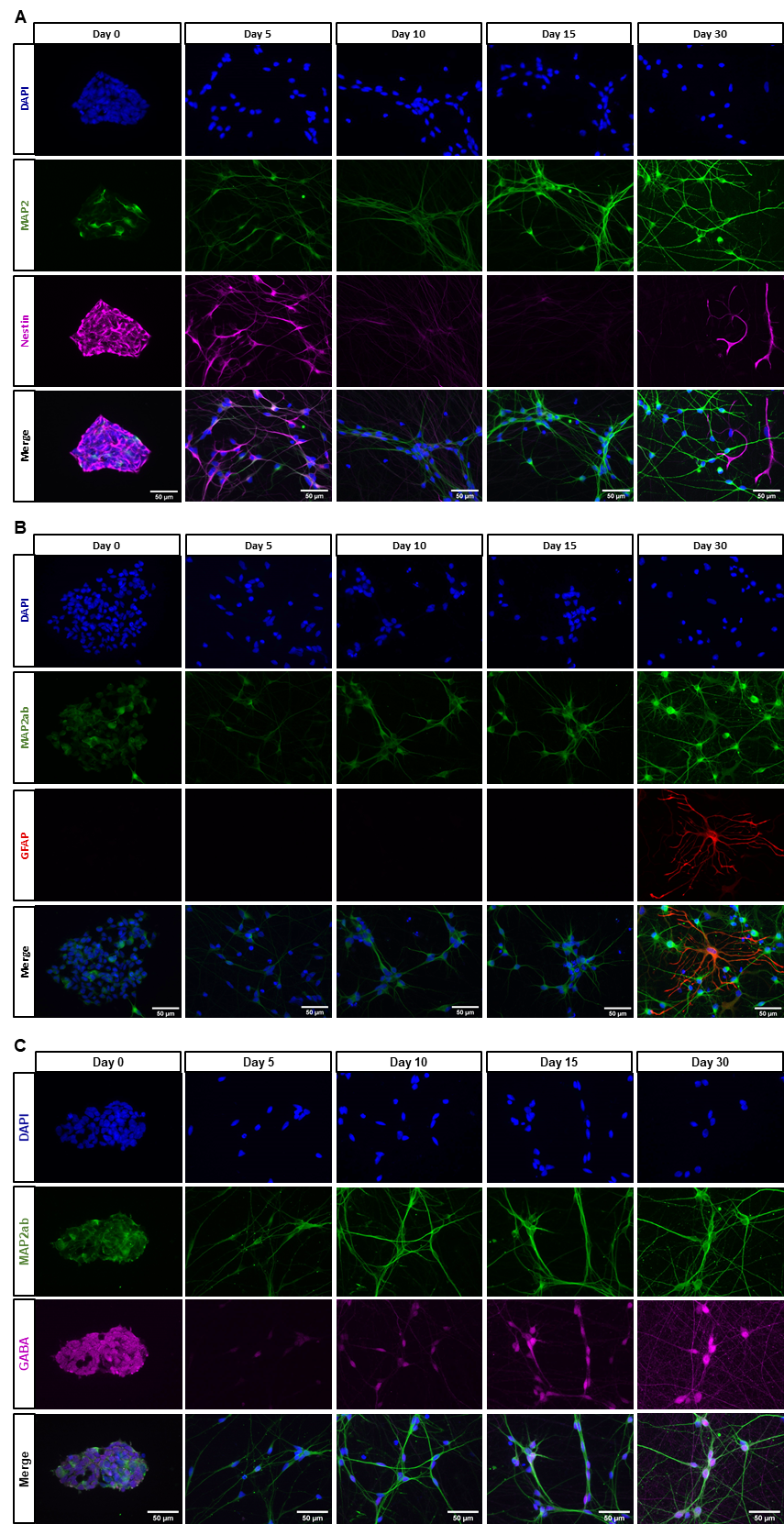


**Supplementary Figure 1.** **Characterization of differentiated neurons over time with immunocytochemistry**. Presented images are taken from an H2/H2 cell line (A) Immunostaining at different time points of differentiation with neural progenitor marker NESTIN and dendritic marker MAP2. At day 0 before induction of differentiation, NESTIN is strongly expressed, while only a few cells express the neuronal marker MAP2. At day 5 of differentiation, most cells are double positive for both markers. While NESTIN expression decreases with further differentiation, MAP2 signal increases. At day 30, most cells are MAP2 positive, but there are also some NESTIN positive cells visible that are MAP2 negative indicating neural progenitor cells that did not differentiate into neurons. (B) Immunostaining at different time points of differentiation with glia marker GFAP and dendritic marker MAP2. From day 0 to day 15, there is no visible expression of GFAP while expression of MAP2 is present from day 0 onwards and increases over time. At day 30, in one cell line there are some GFAP positive cells detectable with an astrocyte morphology. (C) Immunostaining at different time points of differentiation with neurotransmitter GABA and dendritic marker MAP2. At day 5 of differentiation only a few MAP2 positive cells express a very weak GABA signal. At day 10, the signal is still weak but more cells are GABA positive. From day 15 onwards, the signal intensity increases and at day 30 of differentiation, GABA is visible not only in the soma but throughout the neuronal network.


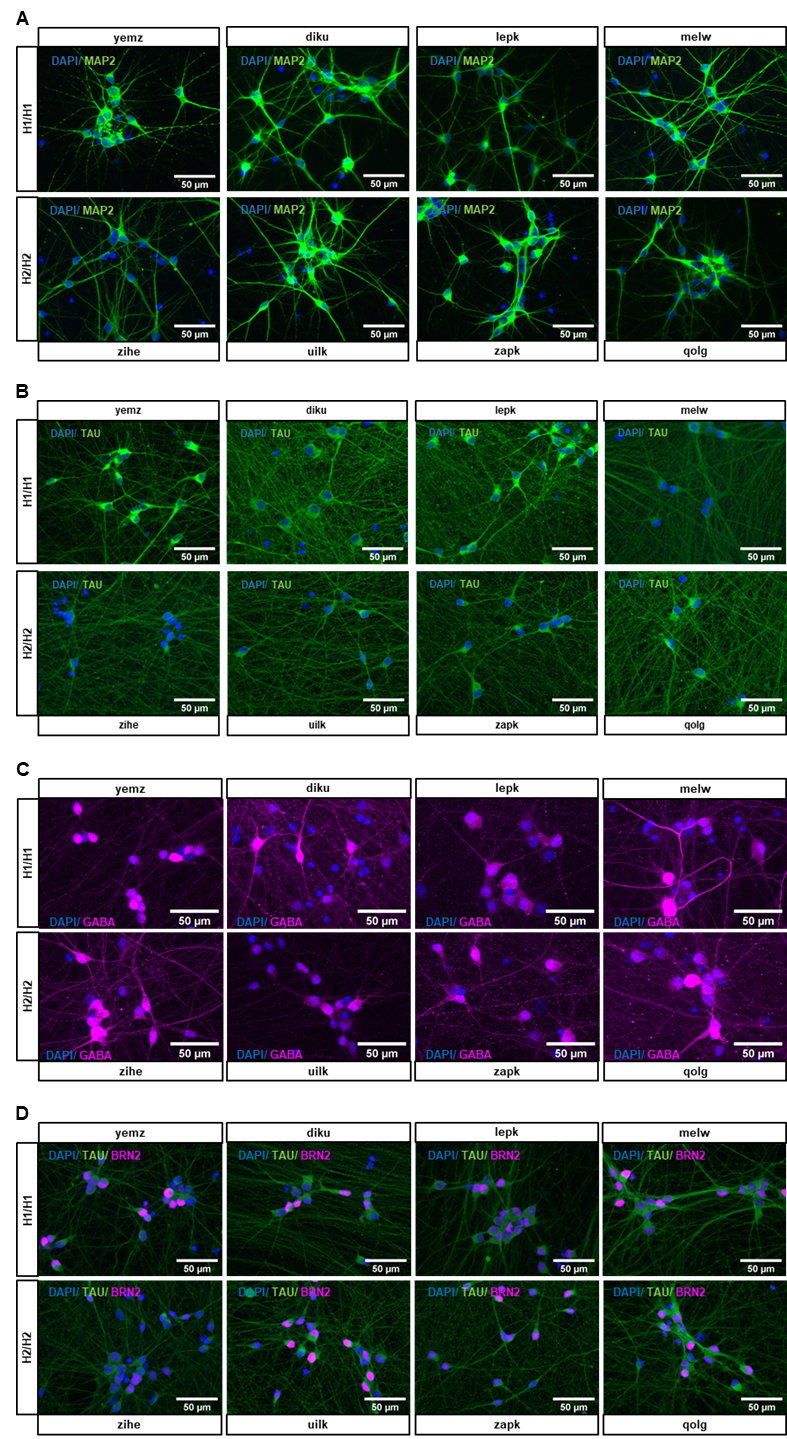


**Supplementary Figure 2.** **Representative pictures at day 30 for all cell lines for neuronal markers.** (A) Immunostaining for dendritic marker MAP2 for all cell lines at day 30 of differentiation shows a comparable staining pattern and cell morphology in all cell lines. (B) Immunostaining for axonal marker TAU at day 30 of differentiation reveals a few differences in TAU signal intensity between cell lines for example in the soma of cells. But the neuronal network looks overall very similar and there are no differences visible that manifest between the *MAPT* haplotype groups. (C) Immunostaining for neurotransmitter GABA at day 30 of differentiation. Some cell lines differ in their GABA signal intensity, but this difference does not manifest between the *MAPT* haplotype groups but are found between cell lines within both groups (D) Immunostaining for cortical marker BRN2 and neuronal marker TAU for all cell lines at day 30 of differentiation shows a comparable staining pattern for both markers in all cell lines.


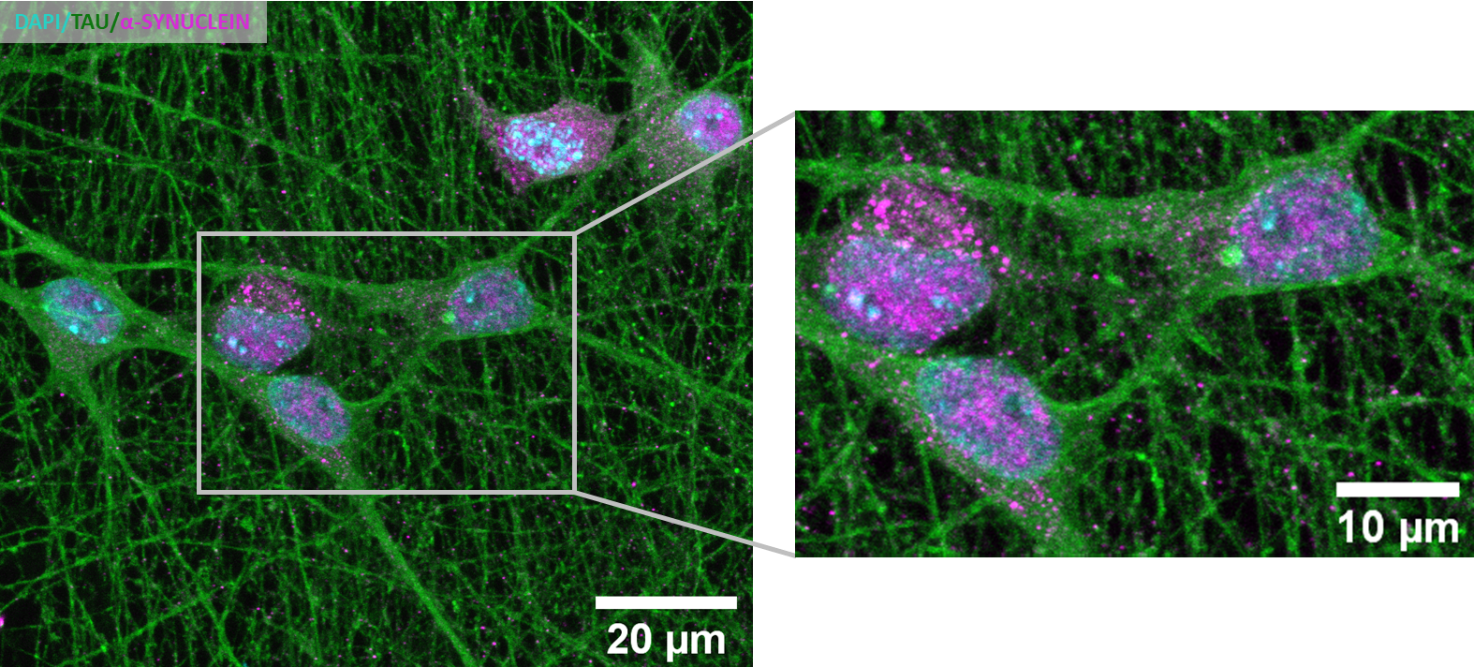


**Supplementary Figure 3.** **Immunostaining of TAU and α-SYNUCLEIN in 30-day old cells.** No clear colocalization of TAU and α-SYNUCLEIN is detectable.


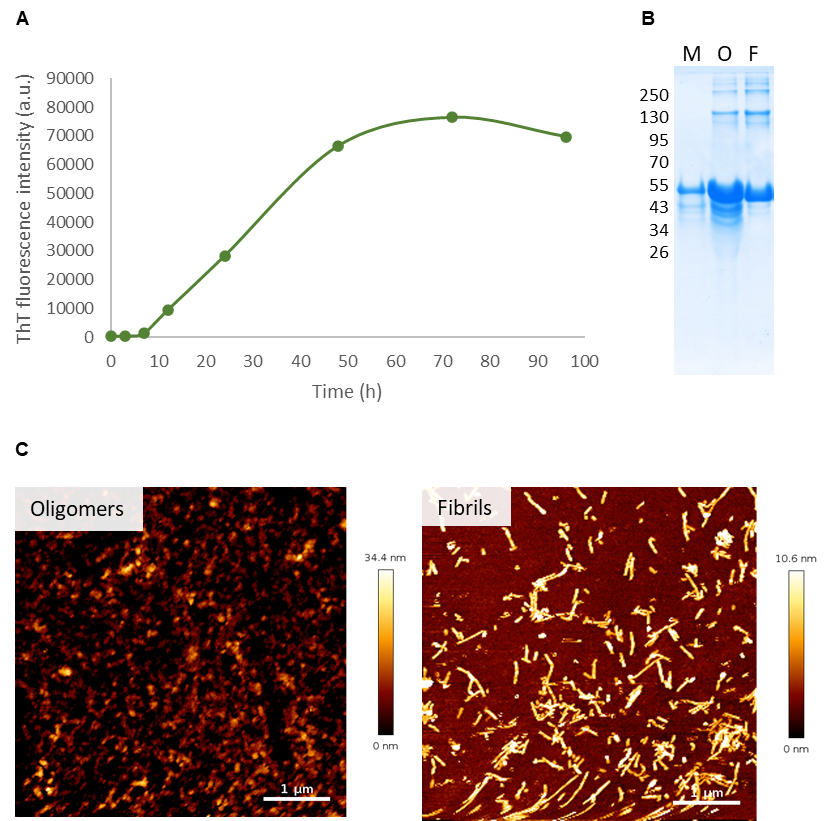


**Supplementary Figure 4.** **Characterization of the TAU fibrillation and aggregated species.** (A) Thioflavin-T showing the kinetics of TAU fibrillation indicating the formation of beta-sheet structures over time. (B) SDS-PAGE for monomeric (M), oligomeric (O) and fibrillar (F) TAU, stained with Coomassie blue. (C) Atomic-force microscopy showing the structure of oligomeric and fibrillar TAU aggregates.


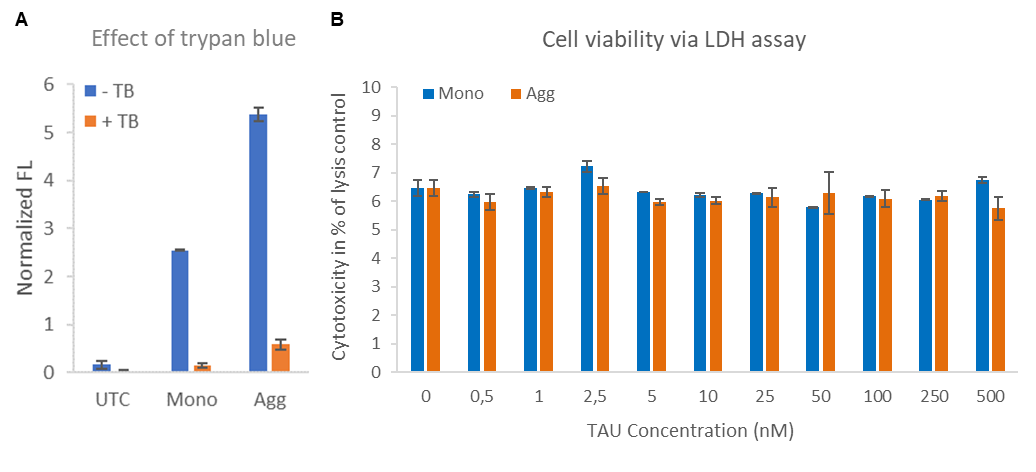
**Supplementary Figure 5.** (A) The impact of trypan blue (TB) on the fluorescence signal of cells treated with TAU monomers (Mono) and aggregates (Agg) after 3 hours of incubation. UTC, untreated control. (B) The LDH-release of cells treated for 48 hours with TAU monomers (Mono) and aggregates (Agg) at increasing concentrations were measured and quantified based on the total induced release of LDH from untreated control cells via triton-X100 (Considered as 100%).


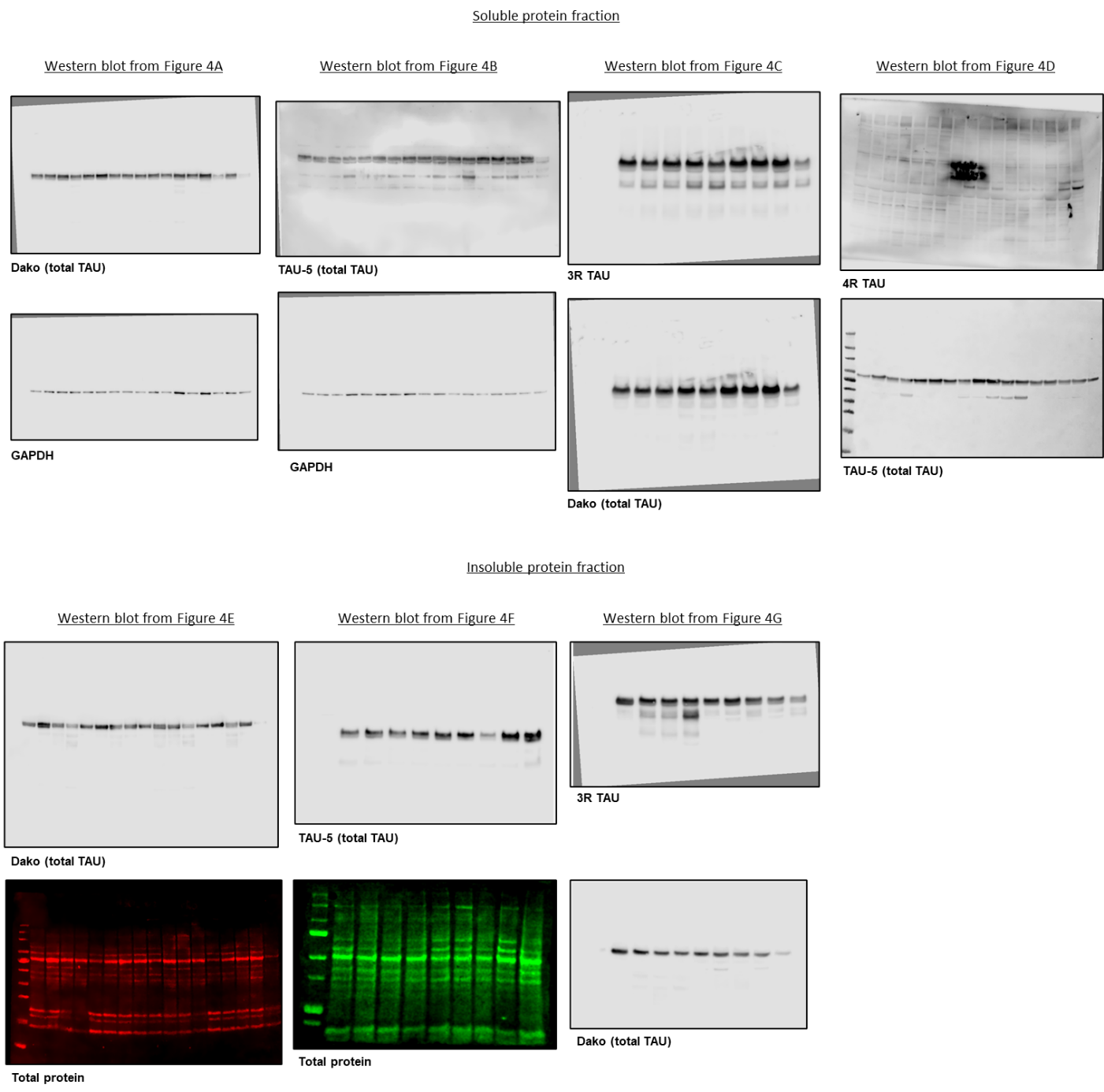


**Supplementary Figure 5.** **Uncropped Western blot images shown in Figure 4**


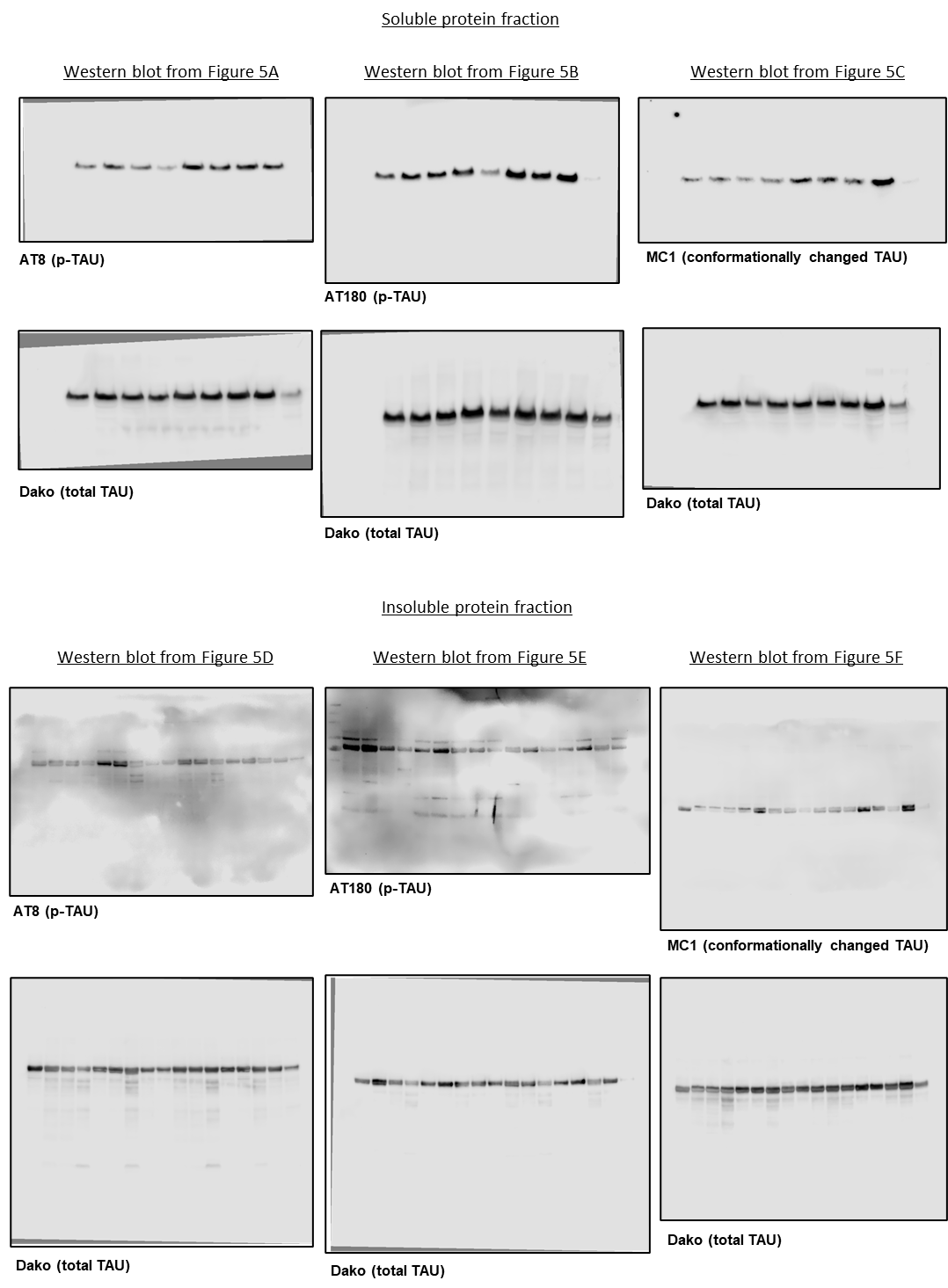


**Supplementary Figure 6.** **Uncropped Western blot images shown in Figure 5**


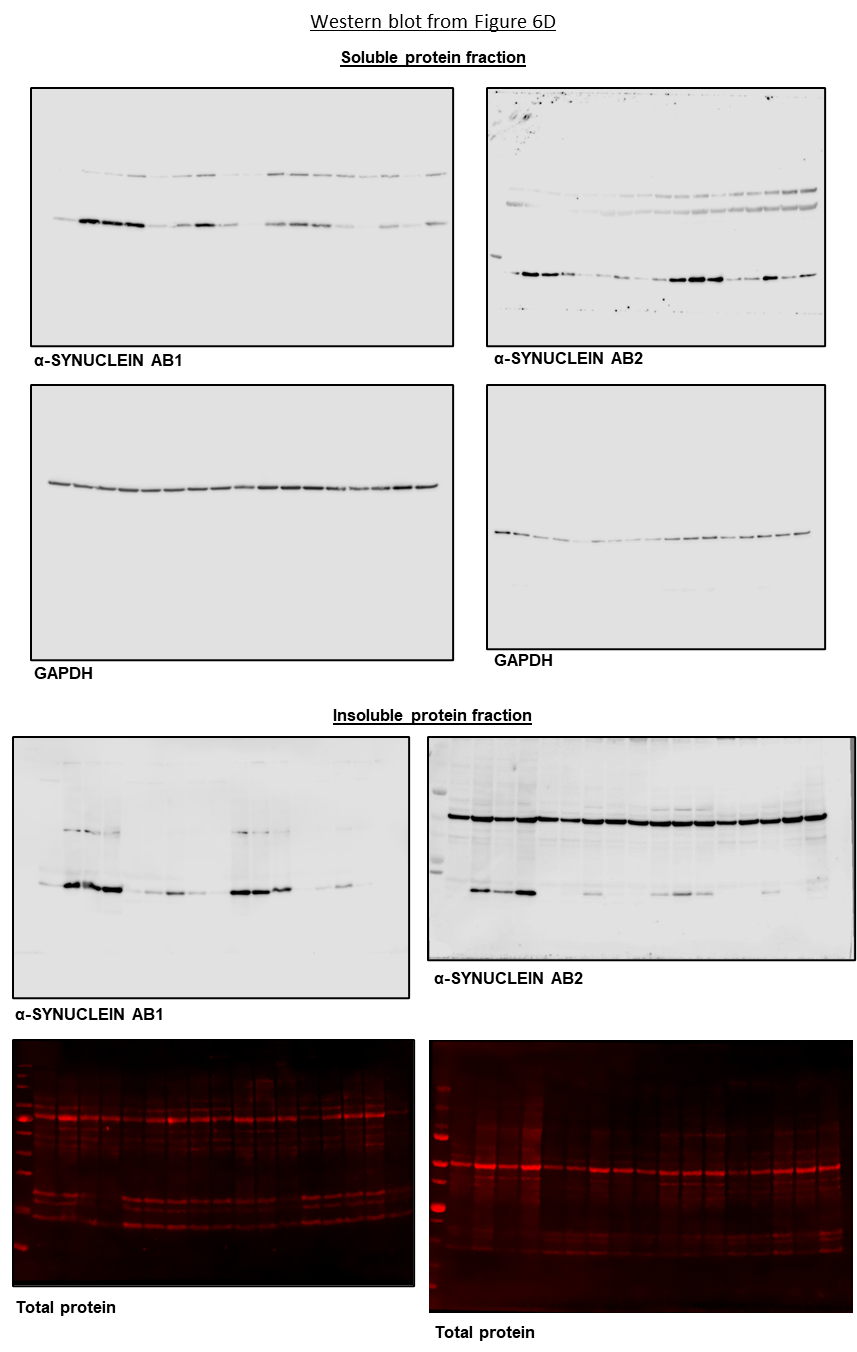


**Supplementary Figure 7.** **Uncropped Western blot images shown in Figure 6**
